# Application of Spectroscopic Methods for Structural Analysis of Chitin and Chitosan

**DOI:** 10.3390/md8051567

**Published:** 2010-04-29

**Authors:** Jolanta Kumirska, Małgorzata Czerwicka, Zbigniew Kaczyński, Anna Bychowska, Krzysztof Brzozowski, Jorg Thöming, Piotr Stepnowski

**Affiliations:** 1 Faculty of Chemistry, University of Gdansk, Sobieskiego 18/19, PL-80-952 Gdansk, Poland; E-Mails: margo@chem.univ.gda.pl (M.C.); zbyszek@chem.univ.gda.pl (Z.K.); annaw@chem.univ.gda.pl (A.B.); gobos@chem.univ.gda.pl (K.B.); sox@chem.univ.gda.pl (P.S.); 2 UFT-Centre for Environmental Research and Sustainable Technology, University of Bremen, Leobener Straße UFT, D-28359 Bremen, Germany; E-Mail: thoeming@uni-bremen.de (J.T.);

**Keywords:** chitin, chitosan, chemical modification, physicochemical parameters, structural analysis using spectroscopic techniques

## Abstract

Chitin, the second most important natural polymer in the world, and its *N*-deacetylated derivative chitosan, have been identified as versatile biopolymers for a broad range of applications in medicine, agriculture and the food industry. Two of the main reasons for this are firstly the unique chemical, physicochemical and biological properties of chitin and chitosan, and secondly the unlimited supply of raw materials for their production. These polymers exhibit widely differing physicochemical properties depending on the chitin source and the conditions of chitosan production. The presence of reactive functional groups as well as the polysaccharide nature of these biopolymers enables them to undergo diverse chemical modifications. A complete chemical and physicochemical characterization of chitin, chitosan and their derivatives is not possible without using spectroscopic techniques. This review focuses on the application of spectroscopic methods for the structural analysis of these compounds.

## 1. Introduction

**Figure 1:**
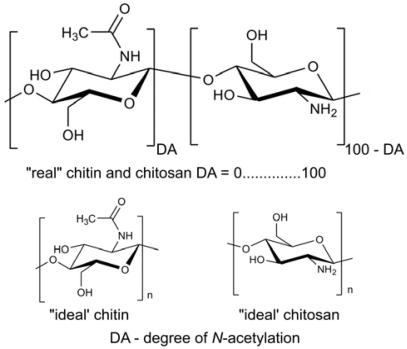
The chemical structures of chitin and chitosan.

Chitin, the second most abundant natural polymer in the world, functions as a natural structural polysaccharide [1]. A major component of the carapaces, crusts and shells of crustaceans such as shrimps, crabs and lobsters, it is also an ingredient of cell walls in fungi and yeast [2]. Its estimated production is 10^10^–10^12^ tonnes per year [3]. Chitin is a linear polymer consisting mainly of β-(1→4)-linked 2-acetamido-2-deoxy-β-d-glucopyranose units and partially of β-(1→4)-linked 2-amino-2-deoxy-β-d-glucopyranose. In this form, chitin is insoluble in water and common organic solvents, dissolving only in specific solvents such as *N*,*N*-dimethylacetamide (DMAc)-LiCl [4], hexafluoroacetone or hexafluoro-2-propanol [5]. When the degree of *N*-acetylation (defined as the average number of *N*-acetyl-d-glucosamine units per 100 monomers expressed as a percentage) is less than 50%, chitin becomes soluble in aqueous acidic solutions (pH < 6.0) and is then called chitosan [6,7]. Thus, chitosan is a collective name for a group of fully and partially deacetylated chitins, but a rigid nomenclature with respect to the degree of *N*-deacetylation between chitin and chitosan has not been defined [8]. The structures of “ideal” chitin and “ideal” chitosan, and the “real” structures of these compounds are shown in Figure 1.

According to the nomenclature proposed by the European Chitin Society (EUCHIS) [9], chitin and chitosan should be classified on the basis of their solubility and insolubility in 0.1 M acetic acid; the soluble material is named chitosan, whereas chitin is insoluble. Chitin and chitosan have a molecular mass of up to several million g/mol. Commercially available chitosan has an average molecular weight ranging between 3,800 and 500,000 g/mol and its degree of *N*-acetylation is 2% to 40% [8,10].

Chitin and chitosan are of commercial interest because of their high nitrogen content (6.89%) and their excellent properties such as biocompatibility, biodegradability, non-toxicity and adsorptive abilities [2,8,11]. These compounds are of very low toxicity: LD_50_ of chitosan in laboratory mice is 16 g/kg body weight, which is similar to that of sugar and salt. In rats, chitosan is safe up to 19% in the diet [12]. As a material that is highly insoluble and has a low chemical reactivity, chitin has limited applicability. Recently, however, chitosan has come back into the spotlight because of its numerous and extensive applications–in biomedicine, waste water treatment, food, cosmetics and the fiber industry [7,8,11,13–17]. Most of the characteristic properties of chitosan are due to the high content of primary amino groups with a pKa of 6.3. At low pH, the positive charge on the-NH_3_^+^ groups converts chitosan to a water-soluble cationic polyelectrolyte; when pH increases to above 6.0 the positive charge on the amino groups is lost and chitosan becomes insoluble [7]. The soluble-insoluble transition of chitosan occurs around pH 6.0–6.5 at the pKa of its primary amino groups. The pKa depends closely on the degree of *N*-acetylation (DA), hence the solubility of chitosan is dependent on DA and the method of *N*-deacetylation [18]. The amino groups are also responsible for several straightforward chemical modifications of chitosan, which predisposes its ongoing development for many applications [19–22]. The improved solubility in water and organic solvents of chemically modified chitin and chitosan has been reported by many scientists [23–25].

Chitin is isolated from the exoskeletons of crustaceans, molluscs, insects and certain fungi, but the main commercial sources of chitin are crab and shrimp shells [2,26]. Depending on the source, chitin occurs in two allomorphs, the α and β-forms, and additionally as γ-chitin, which appears to be a combination of the α and β structures rather than a different allomorph [3]. α-Chitin is by far the most abundant and is usually isolated from the exoskeleton of crustaceans, particularly from shrimps and crabs. β-Chitin can be extracted from squid pens, and γ-chitin from fungi and yeast [27]. β-Chitin is easily converted to α-chitin by alkaline treatment followed by flushing in water [28].

Several techniques to extract chitin from different sources have been published [26,29–31]. Crustacean shells consist of proteins (30–40%), calcium carbonate (30–50%), chitin (20–30%) and pigments (astaxanthin, canthaxanthin, lutein or β-carotene). These proportions vary from species to species and from season to season [32]. The most common method for chemically isolating chitin from crustacean shells involves a number of major steps: the washing, grinding and sieving of raw shells, followed by their demineralization (elimination of calcium carbonate in dilute acidic acid) and deproteinization in aq NaOH or KOH. The use of enzymatic hydrolysis for deproteinization [29] and microorganisms for both demineralization and deproteinization has been also reported [29,33].

Industrially chitin is converted into the more readily applicable chitosan by partial or complete deacetylation of chitin in both the solid (heterogeneous process) and dissolved (homogeneous process) states under alkaline conditions or by enzymatic hydrolysis (using a chitin deacetylase). The source of natural chitin used to produce chitosan affects the production parameters and chitosan preparations. It has been shown that β-chitin is more reactive in *N*-deacetylation than α-chitin [34]. The difference in the semicrystalline morphology of chitin means, for example, that chitosans obtained in a solid-state reaction have a heterogeneous distribution of *N*-acetyl groups along the molecular chains [35]. Differences in the chitosan production process (e.g., temperature, alkali concentration, ratio of alkali solutions to the shells) also mean that chitosan preparations consist of a mixture of chitosans varying in molecular weight and degree of *N*-acetylation. Those produced from chitin may also contain impurities such as heavy metals, protein residues and acid/alkaline residues.

For most of the commercially available chitosans, parameters like weight-average molecular weight (M_W_), polydispersity (M_W_/M_N_), degree of *N*-acetylation (DA), pattern of acetylation (PA) and impurity content (protein, heavy metal) are usually unknown [32,36]. Knowledge of the microstructure of chitosan samples is thus essential for an understanding of the structure–property–activity relationships in them, and special emphasis in this respect is placed on the chitosans used in biomedical applications [32,36,37]. The complete structural characterization of synthesized, biologically active and/or water-soluble derivatives of chitin and chitosan is also very important.

The present paper reviews the applications of spectroscopic methods for the structural analysis and physicochemical characterization of chitin, chitosan and their derivatives. The most important techniques are X-ray spectroscopy, infrared (IR) and UV-Vis-spectroscopy, mass spectrometry (MS), and nuclear magnetic resonance spectroscopy (NMR). The usefulness of these methods for determining and confirming molecular structures, for monitoring reactions and controlling the purity of these compounds is discussed.

## 2. Application of Spectroscopic Methods for Analyzing the Structure and Determining the Physicochemical Properties of Chitin, Chitosan and Their Derivatives

### 2.1. X-ray spectroscopy

X-ray spectroscopy is unarguably the most versatile and widely used means of characterizing materials of all forms [38]. There are two general types of structural information that can be studied by X-ray spectroscopy: electronic structure (focused on valence and core electrons, which control the chemical and physical properties, among others) and geometric structure (which gives information about the locations of all or a set of atoms in a molecule at an atomic resolution). This method encompasses several spectroscopic techniques for determining the electronic and geometric structures of materials using X-ray excitation: X-ray absorption spectroscopy (XAS), X-ray emission spectroscopy (XES), X-ray photoelectron spectroscopy (XPS) and X-ray Auger spectroscopy. Which type of X-ray spectroscopy is employed depends on whether the target information is electronic, geometric or refers to oxidation states: for instance, XAS (first developed by de Broglie) to probe empty states and the shapes of molecules or local structures [39], and XPS (first developed by Siegbahn) to investigate occupied electronic states [40]. X-ray spectroscopy is thus a powerful and flexible tool and an excellent complement to many structural analysis techniques such as UV-Vis, IR, NMR or Raman.

#### 2.1.1. Typical conditions of X-ray measurements

In an X-ray diffraction measurement, a crystal is mounted on a goniometer and gradually rotated while being bombarded with X-rays, producing a diffraction pattern of regularly spaced spots known as reflections [41]. The two-dimensional images taken at different rotations are converted into a three-dimensional model of the density of electrons within the crystal using the mathematical method of Fourier transforms, combined with the chemical data obtained for the sample. If single crystals of sufficient size cannot be obtained, various other X-ray methods, like fiber and powder diffraction, can be applied to record less detailed information.

Clark and Smith in 1937 were the first to make crystal studies of chitin and chitosan using X-ray diffraction (XRD) [42]. They carried out those investigations using a commercial copper-target diffraction tube operated at 30 kV and 25 mA as the X-ray sources, which generated principally Cu-K_α_ lines. The diffraction patterns were recorded on a flat film perpendicular to the beam with the sample 5.0 cm from the film. In later research, the conditions for X-ray measurements of chitin and chitosan were mostly modifications of the ones used by Clark and Smith [42]. For instance, X-ray diffraction measurements were done at 100% relative humidity in a helium atmosphere [43] to avoid the X-ray scattering that led to a “dirty” background on the X-ray film. Present-day X-ray analyses of chitin, chitosan and their derivatives are carried out on advanced X-ray diffractometers.

Apart from traditional X-ray diffraction (XRD), other X-ray techniques for determining chitin/chitosan and their derivatives have been applied: X-ray photoelectron spectroscopy (XPS), the second most popular X-ray spectroscopy technique–for determining the bonding energies of C, O and N atoms on the surface of chitosan and its metal chelate, and for other chitin and chitosan investigations [44–49]; X-ray emission spectroscopy (XES)–perfect for studying the chemical bonding in chitosan and cross-linked chitosan derivatives [50]; X-ray absorption spectroscopy (XAS)–for determining the coordination number of Fe atoms in chitosan-metal complexes [51].

#### 2.1.2. X-ray spectra of chitin and chitosan

Numerous X-ray spectroscopic studies of chitin and chitosan have yielded the diffractive patterns of these compounds [52–59]. However, different sources have characterised these patterns with differently indexed crystalline peaks: these can be labeled either using d, the centre-to-centre spacing of the crystallites, or Miller indices, e.g., (020). It is also very common to describe the diffraction pattern using the values of angles.

**Figure 2:**
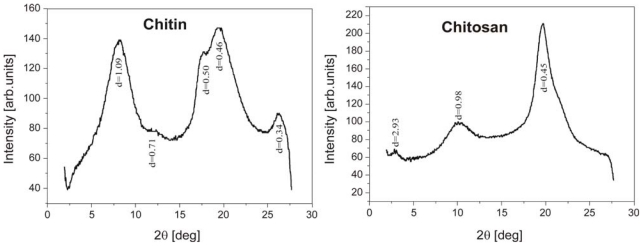
X-ray diffraction spectra of chitin and chitosan fibers. Reprinted from Carbohydrate Polymers 56, 2004, Muzzarelli, C., Francescangeli, O., Tosi, G., Muzzarelli, R.A.A., Susceptibility of dibutyryl chitin and regenerated chitin fibers to deacetylation and depolymerization by lipases, 137–146, Copyright (2010), with permission from Elsevier [56].

Typical spectra of chitin fiber and chitosan fiber are shown in Figure 2 [56]. The spectrum of the former exhibits broad peaks at *d* = 0.34, 0.45, 0.50 and 1.09 nm with a shoulder at 0.71 nm; in the latter the spectral peaks are at *d* = 0.45, 0.88 and 2.93 nm.

The Miller indices of the diffraction peaks characteristic of chitin were (020), (110), (120), (101) and (130) [57,58], although Muzzarelli *et al.* reported the lack of a strong (020) peak for chitin. [59]. In turn, the typical chitin diffraction pattern, given in angle form, showed strong reflections at 2*θ* around 9–10° and 2*θ* of 20–21° and minor reflections at higher 2*θ* values, e.g., at 26.4° and higher [60].

#### 2.1.3. X-ray analysis of chitin and chitosan polymorphs

As already mentioned, the crystal structures of chitosan have been examined since the work of Clark and Smith in 1937 [42]. A great number of diffraction experiments have been undertaken in an attempt to elucidate the molecular geometry of chitosan [42,61–71]. The first X-ray studies showed that the chitosan molecule can adopt at least two different conformations in crystals–a 2-fold [42,61–63] and an eight-fold right-handed helical structure [64,65]. Apart from these two helical conformations, other polymorphs of chitosan have been characterized [66–71]. In 1994 Yui *et al.* determined the detailed crystal structure of the anhydrous form of chitosan by combining X-ray diffraction analysis with a stereochemical model refinement [67]. Chitosan chains crystallized in an orthorhombic unit cell of the following dimensions: *a* = 0.828 nm, *b* = 0.862 nm and *c* (fiber axis) = 1.043 nm. The X-ray diffraction pattern was recorded on the imaging plate, and the intensity of each reflection was estimated by two-dimensional measurement and subsequent background removal. The results showed that the chain conformation of this anhydrous form of chitosan was an extended two-fold helix stabilized by intramolecular O3···O5 hydrogen bonds and that the adjacent parallel chains were connected by O6···N2 hydrogen bonds.

At the same time Mazeau *et al.* examined the conformation and packing of a chitosan polymorph crystallized at a high temperature on the basis of diffraction data [68]. In this kind of polymorph, the chitosan chains form orthorhombic crystals with *a* = 0.807 nm, *b* = 0.844 nm and *c* (chain axis) = 1.034 nm. This chitosan molecule adopted a two-fold helical conformation stabilized by two intramolecular hydrogen bonds, a strong one between O5′ and O3, and a weaker one between O5′ and O6. These two anhydrous forms of chitosan have relatively similar structures.

In 1997 Yui’s group published another paper on the X-ray fiber diffraction method used to determine the crystal structure of a hydrated form of chitosan [69]. The results indicated that the hydrated form of chitosan molecules had a two-fold helical symmetry reinforced by a O3····O5 hydrogen bond with a repeating period of 10.34 Å; this is a structure typical of β (1→4) linked polysaccharides such as cellulose, mannan and chitin.

The following crystalline polymorphs of chitosan have so far been found using X-ray diffraction measurements: the most abundant “tendon-chitosan” [42,69], as well as the “annealed” [63], “1–2”, “L-2” [66], “form I” and “form II” [61] and “8-fold right-handed” forms [64,65]. Apart from the last one, all the polymorphs of chitosan molecules have the extended 2-fold helix configuration; the 8-fold polymorph is unstable and is easily converted into the 2-fold helix [72].

**Figure 3:**
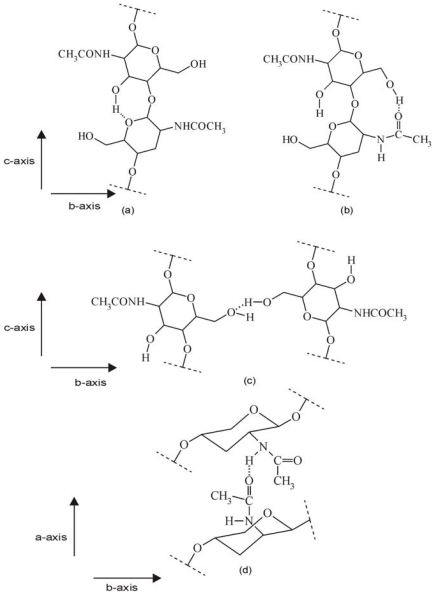
Modes of hydrogen bonding in α-chitin: (a) intrachain C(3′)OH···OC(5) bond; (b) intrachain C(6′_1_)OH···O=C(7_1_) bond; (c) interchain C(6′_1_)O···HOC(6_2_) bond; (d) interchain C(2_1_)NH···O=C(7_3_) bond. Adapted with permission from *Biomacromolecules*. **2000**, *1*, 609–614. Copyright 2010, American Chemical Society [18].

**Figure 4:**
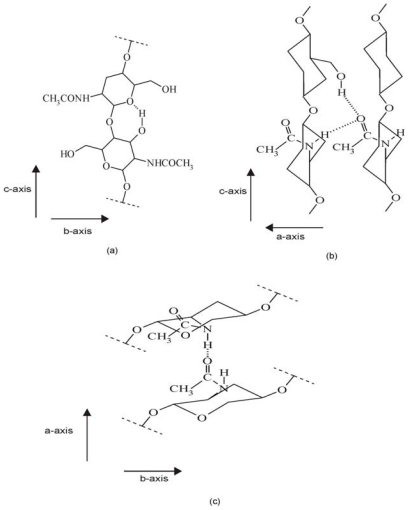
Modes of hydrogen bonding in β-chitin: (a) intrachain C(3′)OH···OC(5) bond; (b) interchain C(2_1_)NH···O=C(7_3_) bond and C(6′_1_)OH···O=C(7_3_) bond (*ac* plane projection); (c) interchain C(2_1_)NH···O=C(7_3_) bond (*ab* plane projection). Adapted with permission from *Biomacromolecules*. **2000**, *1*, 609–614. Copyright 2010, American Chemical Society [18].

Depending on the source, chitin can occur in the α-, β- and γ-forms. The differences among them depend on the arrangement of chains in the crystalline regions [52]. The most abundant and stable form is α-chitin, which Minke and Blackwell studied in detail using XRD in 1978 [45]. These authors determined the α-chitin structure on the basis of intensity data from deproteinized lobster tendon. They discovered that α-chitin chains form orthorhombic crystals with *a* = 0.474 nm, *b* = 1.886 nm and *c* (fiber axis) = 1.032 nm. Additionally, the chains form hydrogen-bonded sheets linked by C=O...H–N bonds approximately parallel to the a axis, and each chain is stabilized by an C(3′)O–H····OC(5) intramolecular hydrogen bond, as in cellulose. These data also indicated that a statistical mixture of CH_2_OH orientations was present, equivalent to half an oxygen on each residue, each forming inter- and intramolecular hydrogen bonds. As a result, Minke and Blackwell found that the α-chitin structure contained two types of amide groups, differing in their hydrogen bonding. In addition, the inability of this polymorph to swell in the presence of water was explained by the extensive intermolecular hydrogen bonding. The modes of hydrogen bonding in α- and β-chitin are illustrated in Figures 3 and 4 [18].

As mentioned above, the differences among chitin polymorphs are due to the arrangement of the chains in the crystalline regions: α-chitin has a structure of antiparallel chains [45], β-chitin has intrasheet hydrogen-bonding by parallel chains [52], and γ-chitin, being a combination of α- and β-chitin [52], has both parallel and antiparallel structures. Because of these differences each chitin polymorph has different properties specific to it. For example, β-chitin is more soluble in and more reactive towards solvents and has a greater affinity towards them; it is also more susceptible to swelling than α-chitin. β-Chitin is also more amenable to *N*-deacetylation than α-chitin. In this case, comparing the diffraction profiles of α- and β- chitin before and after *N*-deacetylation using X-ray spectroscopy seems to be a relatively good solution for distinguishing these forms of chitin. Abdou *et al.* used X-ray diffraction to study two chitin polymorphs, the α- and β-forms, obtained from different sources [60]. The chitin samples were converted into the more soluble chitosan by steeping them in solutions of NaOH of various concentrations and for extended periods of time. The X-ray diffraction patterns of the α-chitin samples and the corresponding hydrolyzed chitosans showed strong reflections at 2*θ* around 9–10° and 2*θ* of 20–21° and minor reflections at higher 2*θ* values at e.g., 26.4° and higher. The chitin bands were sharper than the chitosan bands, even though there was only a slight decrease in the crystallinity percentage. In turn, the X-ray diffraction patterns of the β-chitin samples and their corresponding hydrolyzed chitosans showed that the band at 2*θ* = 9.9° decreased significantly after deacetylation, and that this was followed by a dramatic decrease in the crystallinity percentage. It was therefore concluded that β-chitin is much more amenable to *N*-deacetylation than the α-form. The X-ray diffraction patterns of α- and β-chitin and the corresponding hydrolysed chitosans make them easily distinguishable. The crystallinity index (*CI*) can also be calculated on the basis of X-ray diffractograms. This takes different values for different forms of chitin; for example, Lima showed that *CI* for α-chitin is 28.3% but that for β-chitin it is 20.8% [73].

Many other studies of chitin polymorphs have revealed differences in crystallinity peaks between α-, β- and γ-chitins obtained from various sources [52–55]. For example, Jang *et al.* found crystalline peaks at 9.6, 19.6, 21.1 and 23.7° for α-chitin, at 9.1 and 20.3° for β-chitin, and at 9.6 and 19.8° for γ-chitin [52]. Similarly, Cárdenas *et al.* [53] reported that WAXD patterns of α-chitin (chitins from shrimp, lobster, prawn and king crab) and β-chitin (chitin from squid) exhibited their major characteristic peak at 19.2–19.3° and 18.8° respectively. Kim [54] found that β-chitin from squid pen exhibited crystalline peaks at 9.8° and 19.3°. Yen and Mau [55] found that fungal chitin (γ-chitin) showed two crystalline reflections at 5.4–5.6° and 19.3–19.6°. Irrespective of their origin, the three types of chitin consistently display a major peak at ~19° in their crystallinity structure.

#### 2.1.4. Physicochemical characterization of chitin and chitosan using X-ray diffraction

In 1937, Clark and Smith, in their pioneer X-ray diffraction studies of chitin/chitosan and their derivatives [42], were the first to investigate the physicochemical properties of chitin. They reported the action of hydrochloric acid, lithium thiocyanate and nitric acid on chitin. Their data showed that even at room temperature the ether linkages of chitin were hydrolysed in hydrochloric acid; concurrently, but more slowly, the amide groups were also hydrolysed. In addition, those authors investigated the dispersion of chitin in lithium thiocyanate; at a temperature of 200 °C chitin formed a definite addition compound with lithium thiocyanate, but at lower temperatures only intramicellar swelling was observed. In turn, chitin nitrate was roughly as soluble in hydrochloric acid as the original chitin–no substantial hydrolysis of the acetyl groups had occurred.

Subsequent studies revealed that the properties of chitin and chitosan depended mostly on the degree of *N*-acetylation, molecular weight, polydispersity and crystallinity [31]. Commonly used to measure crystallinity, XRD is also applied to determine the degree of *N*-acetylation of chitin and chitosan [74].

In 1990, Focher *et al.* [75] used XRD to study chitin and postulated the following equation for determining the crystallinity index (*CI*):

(1) $$CI(\%)=[(I_{110}-I_{\text{am}})/I_{110}]\times 100$$

where *I*_110_ (arbitrary units) is the maximum intensity of the (110) peak at around 2*θ* = 19°, and *I*_am_ (arbitrary units) is the amorphous diffraction at 2*θ* = 12.6°. This expression had in fact been employed three years earlier by Struszczyk to determine the *CI* of chitosan [76]. Currently this equation is routinely applied during investigations of chitin, chitosan and their derivatives [73,77,78]. In most cases, *CI* provides information about the crystal state, but it is also very useful for distinguishing α-chitin from β-chitin [73].

On the basis of X-ray powder diffractograms of chitin and chitosan with different degrees of *N*-acetylation, Zhang noted two maximum peaks of the following intensities: one at the (020) reflection and the other at the (110) reflection [74]. He therefore postulated a crystallinity index (*CI*) expressed by two equations:

(2) $${CI}_{020}=[(I_{020}-I_{\text{am}})/I_{110}]\times 100$$

(3) $${CI}_{110}=[(I_{110}-I_{\text{am}})/I_{110}]\times 100$$

Further chitin and chitosan studies indicated that crystallinity could also be assigned from an X-ray diffractogram by dividing the area of the crystalline peaks by the total area under the curve (background area) [60,79,80]. In these calculations, the crystallinity percentage supplied information on relative crystallinity.

A lot of studies have been carried out in which X-ray measurements were applied to determine which parameters affect the crystalline structure of chitin and chitosan and how they do so. In 1991 Ogawa reported an increase in crystallinity with a decrease in the M_W_ of chitosan [71]. One year later,he also determined how chitosan polymorphism and its crystallinity in the membrane depended on the membrane preparation procedure and the molecular weight of the chitosan [43]. XRD measurements demonstrated that when an acetic acid solution of chitosan was dried in air and then soaked in an alkaline solution, both hydrated and anhydrous polymorphs of chitin were present in the resulting membrane. On the other hand, when a highly concentrated solution of chitosan in aqueous acetic acid was neutralized with an alkaline solution, no anhydrous polymorphs were detected in the membrane because drying was incomplete. In another paper from 1993 [81], Ogawa compared the crystallinities of partially *N*-deacetylated chitin (PDC) and partially *N*-acetylated chitosan (PAC) samples with a similar degree of *N*-acetylation and their behavior by heating them in water. He discovered that the *N*-acetylation of pure chitosan is far superior to the solid-state *N*-deacetylation of chitin for producing a less crystalline sample, and in particular, for obtaining a less anhydrous crystal.

The effect of DA on solubility in relation to the crystal structure of deacetylated chitin was also discussed by Cho *et al.* in 2000 [18]. Wide-angle X-ray diffractometry (WAXD) revealed that chitin with ca 72% DA retained the crystal structure of α-chitin with significantly reduced crystallinity and crystallite perfection. The water-soluble chitin with *circa* 51% DA had a new crystal structure resembling that of β-chitin rather than that of either α-chitin or chitosan, suggesting that homogeneous deacetylation converted the crystal structure of chitin from the α- to the β-form.

**Figure 5:**
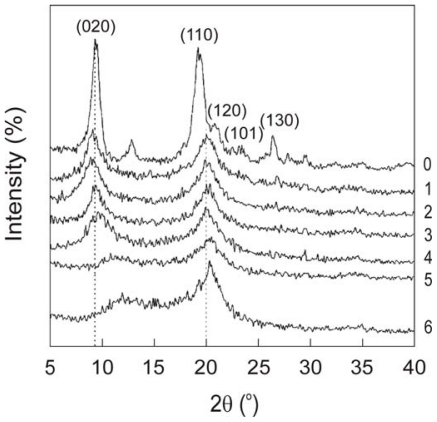
Comparison of X-ray powder diffractograms of chitin and chitosan with different degrees of *N*-acetylation. Figures 0–6 imply different DA (%): 0-83.1, 1-40.6, 2-36.5, 3-41.3, 4-28.6, 5-13.0, 6-7.2. Reprinted from Carbohydrate Research 340, 2005, Zhang, Y., Xue, C., Xue, Y., Gao, R., Zhang, X., Determination of the degree of deacetylation of chitin and chitosan by X-ray powder diffraction, 1914–1917, Copyright (2010), with permission from Elsevier [74].

Using X-ray powder diffraction Zhang *et al.* [74] also tried to look for a relationship between the crystalline state and DA of chitin. Figure 5 presents the XRD patterns of chitin and chitosan with different degrees of *N*-acetylation [74]. These authors noted that the peak of maximum intensity at the (020) reflection diminished together with the decrease in DA and moved to a higher angle. The second intensive peak at the (110) reflection also diminished with the decrease in DA. Consequently, *CI*_020_ decreased linearly with the decrease in DA. This linear relationship between *CI*_020_ and the degree of *N*-acetylation suggested the possible use of XRD for determining DA of macromolecular chitin and chitosan.

As demonstrated, molecular weight and DA are the major parameters significantly influencing the crystal structure of chitin and chitosan, although it has also been reported that the crystallinity index depends on other factors. For example, Seoudi reported that *CI* decreased after chitin was treated with HCl and NaOH [82], and Wada and Saito [57] found that when the biopolymer was heated from room temperature to 250 °C the α-chitin remained structurally stable. Moreover, the influence of alkali-freezing treatment on the solid state structure of chitin was examined by Feng [58]. XRD revealed that during this treatment, the crystal space parameters of chitin changed, and the order of the hydrogen bonds in chitin was modified.

X-ray diffraction was used to measure the elastic moduli E_l_ of the crystalline regions of α-chitin and chitosan [83]. This parameter provided important information on the molecular conformation in the crystal lattice and the mechanism of deformation in the crystalline regions. The data enabled the elastic moduli E_l_ of the crystalline regions in the direction parallel to the chain axis at 20 °C to be assigned as 41 GPa for α-chitin and 65 GPa for chitosan. These E_l_ values, which are low compared to those for cellulose I (138 GPa), were due to the contracted skeletons of α-chitin and chitosan in the crystal lattice. In addition, the elastic moduli calculated from X-ray data showed that the molecular chain of α-chitin in the crystal lattice was mechanically stable from − 190 °C to 150 °C.

#### 2.1.5. X-ray analysis of chitosan salts

Chitosan has a regular distribution of aliphatic primary amino groups along its chain. These produce salts when the molecule reacts with inorganic or organic acids. Ogawa and Inukai used XRD to study several crystallized inorganic acid salts of chitosan [64]. On the basis of X-ray fiber diffraction, these authors suggested that chitosan acid salts took up two different conformations. One, which they called “type I salt”, retained the extended two-fold helix of the unreacted chitosan molecule, although they were different crystals. The second one, the “type II salt”, had an eight-fold helical conformation in the crystal. The salts forming with HNO_3_, HBr and HI took up the former structure, and those with HF, HCl, and H_2_SO_4_ the latter one. Crystals of type I salts were anhydrous, whereas those of type II were hydrated. Despite the different anion sizes, all the type II salts gave fiber patterns that were very similar to each other. They crystallized in a monoclinic unit cell with a helical repetition of 4.073 nm. The chirality of the eight-fold helix was right-handed, since their fiber patterns were very similar to those obtained by Cairns *et al.* [65].

Apart from inorganic salts, chitosan can easily form organic salts, for example, when it reacts with L-ascorbic acid. In 1996 Ogawa carried out an X-ray study of the chain conformation of the ascorbic acid salt of chitosan [84]. He determined that both l- and d-ascorbate chitosan salts retained the extended 2-fold helical conformation of the unreacted chitosan chain and that both crystals were anhydrous. In view of these results, he classified them as type I salts of the chitosan acid salts [64], although during the preparation of the l- and d-ascorbates of chitosan, that author discovered that d-ascorbic acid displayed a higher reactivity towards chitosan than the l-isomer. Those differences in reactivity between l- and d-ascorbic acids could be connected with the optical resolution of ascorbic acid.

Chitosan salts were also examined in 1999 by Kawada *et al.* [72]. They studied the spontaneous water-removing action of acid by preparing chitosan salts of many different (monocarboxylic, inorganic and organic) acids, and examined their structures using X-ray diffraction. The results indicated that the temperature required for salt formation depended on the hydrophobicity of the acid; for instance, the chitosan formic acid salt could be prepared at room temperature, whereas the formation of the propionic acid salt had to be carried out at 4 °C. Moreover, type II salts of monocarboxylic acids, the hydrated crystals of chitosan, could be dehydrated even at room temperature without any loss of orientation or decomposition of the chitosan specimen.

#### 2.1.6. X-ray analysis of chitosan derivatives

Chitin and chitosan are a family of polymers with highly variable chemical and physical properties. These compounds and their derivatives have at least 200 potential and current applications [32] in the biomedical, food, biotechnological, agricultural and cosmetics industries.

Muzzarelli and co-workers found that chitosan exhibited good adsorption selectivity towards some transition and post-transition metal ions from aqueous solution [85]. A chitosan-metal complex dissociates easily when the pH is lowered; therefore, chitosan is very often used in the recovery of useful transition metals from waste. Ogawa and Oka reported on the behavior of chitosan-metal complex formations examined by X-ray diffraction [86]. The unit cells of all the salt complexes studied were orthorhombic, although the cupric salt complexes showed some unindexed reflections. Their lattice parameters and the number of water molecules in the cell depended on the counteranions of the metal salt and not on the metal ion. The ratio of glucosamine residues to metal salts was 2:1. On the basis of the fiber diffraction patterns of various chitosan-transition metal salt complexes, these authors postulated a coordination mode which they named the “pendant model”. This model had already been put forward by Ogawa *et al.* back in 1984, but unequivocal experimental evidence to support it was lacking [70]. In this “pendant model” metal anions were coordinated to the amino groups of the chitosan chain like a pendant. Additionaly there was another contrasting model called “the bridge model”, in which metal ions were coordinated with four nitrogen atoms of the intra- and inter-chitosan chains [87]. In spite of these differences, chitosan exhibits a high affinity for metal ions, a property that has been used to recover transition metals from waste water. At present, the adsorption properties of metal ions on chitin and chitosan derivatives are still routinely examined by X-ray diffraction [49,88–90].

The majority of current studies on chitin and chitosan are seeking to discover new derivatives with unusual properties and different potential applications [56,91–97]. X-ray measurements are still very often applied to characterize most of these new derivatives. For example, chitosan-based nanocomposite films, containing chitin nanocrystals as functional components, were successfully prepared and cross-linked using glutaraldehyde [97]. XRD showed that chitin nanocrystals retained their crystalline morphology in the nanocomposites before and after cross-linking, and that chitosan also retained its amorphous characteristics in the nanocomposites. Another example, novel chitosan/gelatin membranes were prepared using a suspension of chitosan hydrogel mixed with gelatin [95]. XRD studies showed that the chitosan and gelatin in these membranes are compatible and interact well with each other. In addition, the incorporation of gelatin reduced the crystallinity of chitosan. Finally, natural rubber/chitosan blends were studied by XRD analysis [91], the measurements indicating that vulcanization enhanced the crystallinity.

#### 2.1.7. Other X-ray techniques used in chitin and chitosan analysis

As mentioned at the beginning of this section, X-ray spectroscopic techniques other than the traditional X-ray diffraction measurements have been successfully used to analyze chitin, chitosan and their derivatives.

X-ray photoelectron spectroscopy (XPS) is usually used to determine the bonding energies of C, O and N atoms on the surface of chitosan and its metal chelates, although this is not its only use [44–49]. For example Matienzo and Winnacker [46] presented high-resolution C1s, N1s and O1s XPS spectra for a chitosan film coated on an Al-silicon surface. The films were treated in either an oxygen plasma environment or under UV/ozone irradiation. XPS data showed that hydroxyl and amine entities participated only minimally in the modification. In addition, deposition of chitosan films onto Al-coated silicon wafers produced films with a more ordered chitosan structure. Surface analysis of modified films by XPS also indicated that neither the hydroxyl groups nor the amine segments appeared to participate in surface degradation reactions by either UV/ozone or oxygen plasma during the exposure times chosen for those studies. XPS also provided information regarding the forms of species absorbed on the polymer [48]. For example, a study of the interactions of Cu^2+^, Mo^4+^ and Cr^3+^ with chitosan beads, cross-linked chitosan beads and chitosan flakes revealed that the adsorption of Mo^4+^ and Cr^3+^ on chitosan flakes and beads was followed by reduction of the Mo^4+^ and Cr^3+^. Another, example of the use of XPS was presented by Veleshko *et al.* [47]. They examined the complexation between the uranyl group U(VI) and chitosan by means of X-ray photoelectron spectra. The results showed that the interaction of chitosan with the uranyl group yielded complexes containing the nitrogen atom of the amino group and, very probably, oxygen atoms from the chitosan ring and free hydroxyl groups in the equatorial plane.

X-ray emission spectroscopy (XES), also known as X-ray fluorescence spectroscopy (XFS), is a very sensitive probe for examining the local electronic structure and chemical bonding of the emitting atoms. This X-ray technique was used by Kurmaev *et al.* in 2002 [98] to study the chemical bonding in chitosan and chitosan cross-linked with ethylene glycol diglycidyl ether (EGDE). These authors concluded that the changes in the width of resonantly excited O Kα XES were due to site-selective excitation of oxygen atoms belonging to different functional groups (OH and –O–). Comparison of the nitrogen Kα spectra of unmodified and cross-linked chitosan proved that the preferred structural model was the one according to which EGDE was linked via the hydroxyl group.

### 2.2. Infrared spectroscopy

Infrared (IR) spectroscopy is one of the most important and widely used analytical techniques available to scientists working on chitin and chitosan. It is based on the vibrations of the atoms of a molecule. The infrared spectrum is commonly obtained by passing infrared electromagnetic radiation through a sample that possesses a permanent or induced dipole moment and determining what fraction of the incident radiation is absorbed at a particular energy [99]. The energy of each peak in an absorption spectrum corresponds to the frequency of the vibration of a molecule part, thus allowing qualitative identification of certain bond types in the sample. An IR spectrometer usually records the energy of the electromagnetic radiation that is transmitted through a sample as a function of the wavenumber or frequency. Nowadays, the total spectrum is analyzed by an interference process and converted into the frequency or wavenumber range by means of a mathematical process known as the Fourier transform. Fourier-transform infrared (FTIR) spectroscopy has dramatically improved the quality of infrared spectra and minimized the time required to obtain data [99,100]. Progress in modern infrared spectroscopy is reviewed in literature [101,102].

#### 2.2.1. Typical conditions for the FTIR spectroscopic analysis of chitin, chitosan and their derivatives

FTIR spectra are usually recorded in the middle infrared (4000 cm^−1^ to 400 cm^−1^) with a resolution of 4 cm^−1^ in the absorbance mode for 8 to 128 scans at room temperature. The samples for FTIR analysis are prepared by grinding the dry blended powders with powdered KBr, often in the ratio of 1:5 (Sample: KBr) and then compressed to form discs. Spectra are sometimes measured using a deuterated triglycerinesulphate detector (DTGS) with a specific detectivity of 1 × 10^9^ cmHz^1/2^ w^−1^ [103] or on films using an attenuated total refraction (ATR) method in an IR spectrometer [104–106]. Diffuse Reflectance Infrared Fourier-Transform (DRIFT) spectroscopic analysis is also applied [107].

#### 2.2.2. Physicochemical characterization of chitin and chitosan using infrared spectroscopy

**Figure 6:**
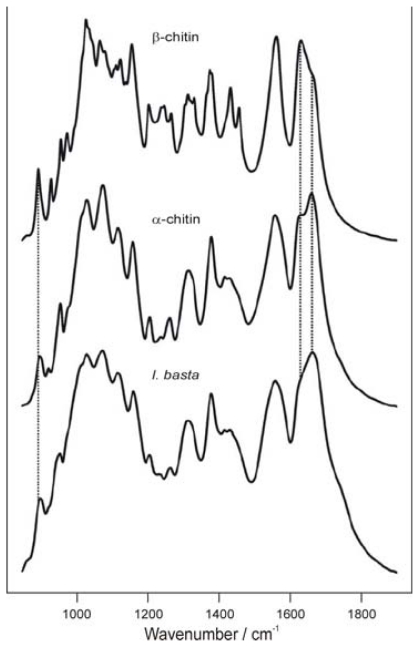
FTIR spectra of β-chitin from *T. rotula*, α-chitin from crabs and *I. basta* chitin after NaOH and H_2_O_2_ treatment. Dashed vertical lines are drawn to mark characteristic differences between α- and β-chitin. Reprinted from Journal of Structural Biology 168, 2009, Brunner, E., Ehrlich, H., Schupp, P., Hedrich, R., Hunoldt, S., Kammer, M., Machill, S., Paasch, S., Bazhenov, V.V., Kurek, D.V., Arnold, T., Brockmann, S., Ruhnov, M., Born, R., Chitin-based scaffolds are an integral part of the skeleton of the marine demosponge *lanthella basta*, 539–547, Copyright (2010), with permission from Elsevier [110].

As already mentioned, natural chitin occurs mainly as α- and β-chitin. The description and interpretation of the infrared spectra of these two forms of chitin have been published by many scientists [108–110]. By way of example, the spectra of α- and β-chitin, and the *Ianthella basta* scaffold after NaOH treatment and H_2_O_2_ purification are shown in Figure 6 [110].

The spectra of α- and β-chitin display a series of narrow absorption bands, typical of crystalline polysaccharide samples. The C=O stretching region of the amide moiety, between 1700 and 1500 cm^−1^, yields different signatures for α- and β-chitin. For α-chitin, the amide I band is split into two components at 1660 and 1630 cm^−1^ (due to the influence of hydrogen bonding or the presence of an enol form of the amide moiety [109–111]), whereas for β-chitin it is at 1630 cm^−1^ (Figure 6). The amide II band is observed in both chitin allomorphs: at 1558 cm^−1^ for α-chitin and 1562 cm^−1^ for β-chitin [110]. Another characteristic marker is the CH deformation of the β-glycosidic bond. This band shifts from 890 cm^−1^ in β-chitin to 895 cm^−1^ in α-chitin. Infrared spectra of β-chitin reveal two additional bands for CH_x_ deformations at about 1455 and 1374 cm^−1^ and a greater number of narrower bands in the C–O–C and C–O stretching vibration region (1200–950 cm^−1^) not observed in α-chitin. As shown in Figure 6, the FTIR spectrum of the chitin isolated from *I. basta* confirmed the finding that this chitin resembles α-chitin more closely than β-chitin [110], demonstrating that FTIR can be used to determine chitin allomorphs.

FTIR spectroscopy has been used to characterize not only isolated chitin [110] but also the source of chitin, e.g., in two species of black coral, *Antipathes caribbeana* and *A. pennacea* [112]. Although FTIR absorption spectra of the natural samples (without deproteinization) showed similar distribution patterns for both species of coral, and confirmed the presence of chitin in both species, small differences were observed (e.g., the intensity of the IR absorption bands in *A. caribbeana* was stronger). The absence of a free hydroxyl in the hydroxymethyl groups CH_2_OH in *A. caribbeana* (determined by FTIR analysis) indicated that the chitin chains were organized in sheets, where they were hydrogen-bonded to adjacent chains, a situation that favors a denser fiber packing of chitin. This means that the FTIR measurements permitted an explanation of why natural *A. caribbeana* coral was harder to pulverize and required a longer deproteinization time than *A. pennacea*. The presence of chitin in polyplacophoran sclerites was also confirmed by IR [113].

**Figure 7:**
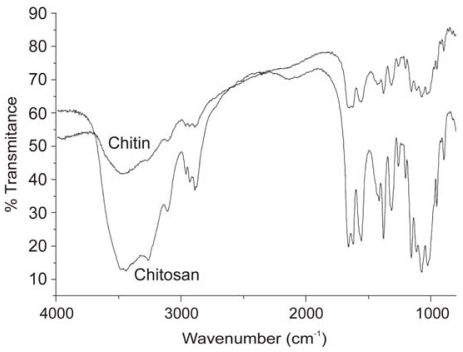
FTIR of chitin (A) and chitosan (B) produced from silkworm pupae; range: 1400–1700 cm^−1^. Reprinted from Carbohydrate Polymers 64, 2006, Paulino, A.T., Simionato, J.I., Garcia, J.C., Nozaki J., Characterization of chitosan and chitin produced from silkworm crysalides, 98–103, Copyright (2010), with permission from Elsevier [114].

FTIR spectroscopy has also been used to compare the yield and purity of chitin isolated from pupae of the silkworm (*Bombyx mori*) using two methods of extraction: an open reactor and 1 h of an acidic reaction, and extraction in a closed reactor within 24 h of a basic reaction [114]. The efficiency of chitosan production by the *N*-deacetylation of chitin was also investigated by IR in this work. During the *N*-deacetylation of chitin, the band at 1655 cm^−1^ gradually decreased, while that at 1590 cm^−1^ increased, indicating the prevalence of NH_2_ groups. The spectra of chitin and chitosan obtained from the *N*-deacetylation of chitin using a solution of NaOH (40 wt%) in the presence of NaBH_4_ for 5 h are presented in Figure 7.

The band at 1590 cm^−1^ displayed a greater intensity than the one at 1655 cm^−1^ and demonstrated the effective deacetylation of chitin. FTIR analyses were also used to find the optimal conditions for the *N*-deacetylation of chitin whiskers (the alkali concentration and the treatment time) using a microwave technique [115].

Prashanhi *et al.* [116] applied IR spectroscopy to observe the changes occurring in the crystallinity and polymorphic nature of chitosan as a function of the *N*-deacetylation of chitin under different conditions: uncontrolled conditions (chitosan A), under an N_2_ atmosphere (chitosan B), and with thiophenol (chitosan C). The FTIR spectra of chitosans A, B, and C were similar to each other, but there were subtle differences in the absorption intensities. Apart from the expected decrease in the band at 1665 cm^−1^ (amide I), the vibrational mode of amide II at 1550 cm^−1^ for chitin appeared at 1604 cm^−1^, 1598 cm^−1^ and 1592 cm^−1^ for chitosan A, B and C respectively [116]. In none of these spectra were there any sharp absorptions at *circa* 3500 cm^−1^, which confirms that the hydroxyl groups in positions C2 and C6 of the chitosans are involved in intra- and intermolecular hydrogen bonds. The region above 3000 cm^−1^ was centred at 3395 cm^−1^ in chitosan A, at 3407 cm^−1^ in chitosan B and at 3419 cm^−1^ in chitosan C; the shift to the higher frequency demonstrated a higher-order structure for these three chitosans. The CH_2_ stretching bands of chitosan B around 1425 cm^−1^ were more intense than those of chitosans A and C. Furthermore, the FTIR spectra exhibited a progressive weakening of the bands at 3265 cm^−1^ and 3100 cm^−1^ during *N*-deacetylation, and the A_1382_/A_2920_ cm^−1^ ratios of 0.65, 0.56 and 0.46 indicated a higher order structure of the chitosans prepared with thiophenol than those prepared under an N_2_ atmosphere. The ratio of the band intensities at 1379 and 2900 cm^−1^ was also used to estimate the crystallinity of chitin and chitosan by Focher *et al.* [75] and Wu *et al.* [117], whereas Prashanth and Tharanathan used the sharp absorption peak around 618 cm^−1^ [118].

The impurities in chitosan preparation were determined by FTIR analysis [119]. The FTIR spectra of control chitosan and low-molecular-weight water-soluble chitosan (LMWSC) prepared in this study were compared to establish the contaminants and prove the synthesis of LMWSC. In the LMWSC spectrum the carboxyl group absorption band derived from lactic acid and impurities formed during enzyme degradation disappeared or significantly decreased.

FTIR spectroscopy has been employed to measure the critical concentration of two chitooligosaccharides form a lyotropic liquid crystalline phase in formic acid (*C_1_*) [120]. Strong interactions between sugar chains and solvent were revealed by the widening of bands attributed to the –OH, –NH, –NHCO– of the chitooligosaccharide, including the C=O of formic acid. FTIR measurements of the shift of seven bands–1. –NH_2_, –OH (3390–3418 cm^−1^), 2. C=O of formic acid (1716–1724 cm^−1^), 3. amide I (1626–1633 cm^−1^), 4. amide II (1520–1531 cm^−1^), 5. 6. C_3_–OH (double peaks, 1178–1189 cm^−1^ and 1148–1153 cm^−1^), 7. C_6_–OH (1073–1074 cm^−1^)–enabled the C_1_ values of these chitooligosaccharides to be established.

#### 2.2.3. Determination of the degree of *N*-acetylation of chitin and chitosan using infrared spectroscopy

The degree of *N*-acetylation is one of the most important chemical parameters capable of influencing the performance of chitosan and chitin in many of their applications [2,7,121]. Of the various analytical techniques developed for DA determination [122], infrared spectroscopy is at the centre of attention. A convenient and relatively quick technique, it allows the DA values of chitin/chitosan to be determined on the basis of absorption ratios, also in the solid state [123]. Several procedures using different absorption ratios have already been proposed for determining DA for chitin and chitosan samples [124–130]. A review article summarizing the latest literature information on DA determination by IR spectroscopy for chitin and chitosan was published by Kasaai [131]. In that paper, various IR procedures were compared for their performances and limitations, advantages and disadvantages, and different factors affecting the experimental results were discussed, as were the validity data of DA measurements by FTIR spectroscopy. In view of this, the present review will discuss only general information on DA determination by IR.

DA can be determined by IR techniques in the following ways:

- Determination of the *A_M_/A_R_* ratio, where *A_M_* is the intensity of the characteristic band of *N*-acetylation, which is a measure of the *N*-acetyl or amine content, and *A_R_* is the intensity of a reference band that does not change with different DA values. The DA parameter of unknown samples can be established by comparing the determined *A_M_/A_R_* values with similar ratios of a few reference samples of known DA.
- Drawing a calibration curve by plotting the absorption ratio of chitin/chitosan samples of known DA *versus* their DA as established by IR or a reference method such as ^1^H NMR spectroscopy. The DA values of unknown samples can then be estimated from the calibration curve.
- Statistical evaluation of several absorption band ratios [131,132].

**Figure 8:**
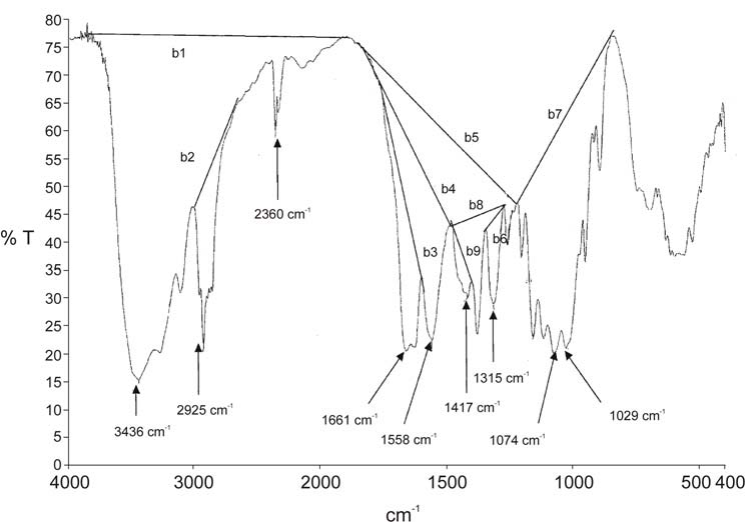
IR spectrum of chitin. Representation of the different baselines mentioned in the literature. Reprinted from Polymer 42, 2001, Brugnerotto, J., Lizardi, J., Goycoolea, F.M., Argüelles-Monal, W., Desbrières, J., Rinaudo, M., An infrared investigation in relation with chitin and chitosan characterization, 3569–3580, Copyright (2010), with permission from Elsevier [129].

IR techniques require choosing an appropriate band measure, an appropriate reference band, and drawing a good base line, necessary for measuring the intensity of absorption. The amide I bands at 1655 cm^−1^ (sometimes together with the amide I band at 1630 cm^−1^) or the amide II band at 1560 cm^−1^ are used as the characteristic band(s) of *N*-acetylation. Among the postulated internal reference bands are the OH stretching band at 3450 cm^−1^ [125,133], the C-H stretching band at 2870–2880 cm^−1^ [134], the –CH_2_ bending centred at 1420 cm^−1^ [129], the amide III band at 1315–1320 cm^−1^ [135], the anti-symmetric stretching of the C-O-C bridge at around 1160 cm^−1^ [136], the skeletal vibrations involving the C-O-C stretching bands at 1070 or 1030 cm^−1^ [127,128] and the band at 897 cm^−1^ (C-O-C bridge as well as glycosidic linkage) [137]. The different baselines suggested in the literature [129] are presented in Figure 8.

**Figure 9:**
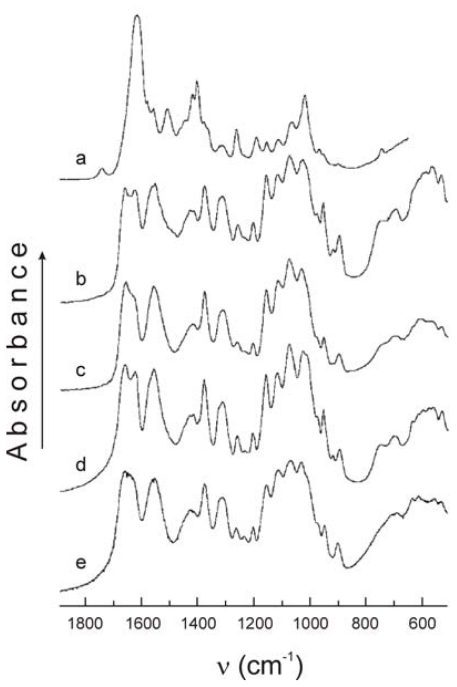
Comparison of IR spectra (shown as absorbance) of α- and β-chitin recorded using different sampling techniques. For α-chitin: (a) ATR on film, (b) DRIFT on powder, (c) Standard transmission on film, (d) Standard transmission on KBr pellet. For β-chitin: (e) Standard transmission on KBr pellets. Reprinted from Polymer 42, 2001, Brugnerotto, J., Lizardi, J., Goycoolea, F.M., Argüelles-Monal, W., Desbrières, J., Rinaudo, M., An infrared investigation in relation with chitin and chitosan characterization, 3569–3580, Copyright (2010), with permission from Elsevier [129].

Many different absorption band ratios, such as A_1560_/A_2875_, A_1655_/A_2875_, A_1655_/A_3450_, A_1320_/A_3450_, A_1655_/A_1070_, A_1655_/A_1030_, A_1560_/A_1160_, A_1560_/A_897_ and A_1320_/A_1420_, have been used to determine DA by FTIR spectroscopy [131]. The different calibration curves proposed in the literature have different baselines and different characteristic bands for measuring the *N*-acetyl content. Moreover, the validity data of these calibrations depend on the absolute technique used to measure DA, and the conditions under which IR spectra are measured may also have a strong influence on the accuracy of DA values. The IR spectra (shown as absorbance) of α-chitin and β-chitin recorded using different sampling techniques are compared in Figure 9 [129].

The ATR spectrum recorded on the film is shown in Figure 9a. This revealed a very low resolution following the collection of 64 scans recorded in typical fashion. The amide I bands (doublet at 1655 and 1625 cm^−1^) could not be resolved as they were focused into a single band when compared to the standard transmission spectrum of α-chitin obtained in a KBr pellet (Figure 9d). Also, the characteristic band at 1320 cm^−1^ was of very low intensity. By contrast, DRIFT analysis of α-chitin (mixed with HBr) on powder produced a spectrum (Figure 9b) of much better resolution; the amide I band was split into two components. In the transmission spectrum of α-chitin film (Figure 9c) the amide band showed a well-defined peak at 1650 cm^−1^ with a minor shoulder at 1625 cm^−1^. Knowledge of the source of chitin/chitosan, its nature, water content and level of impurities is also important for the accurate calculation of DA values.

Near infra-red (NIR) spectroscopy has also been applied to determine DA [138,139]. The spectra were recorded from 9090–4000 cm^−1^ and second-derivative spectra were used to determine DA; d-glucosamine and d-glucosamine hydrochloride were chosen as model compounds. A reference curve was constructed by plotting the predicted DA value (using NIR data) *versus* DA determined by ^1^H NMR spectroscopy.

As shown here, choosing an appropriate method among the various IR procedures is a difficult task for researchers.

IR techniques are also employed for the quantitative evaluation of DA, for example, during the *N*-deacetylation of chitin by hot alkali [140], the formation of *N*-acetyl chitosan gel [141], the γ-irradiation of chitosan powder [142] or the synthesis of organic-soluble acetylated chitosan [143].

#### 2.2.4. FTIR analysis of chitin and chitosan derivatives

The growing interest in the chemical modification of chitin and chitosan to improve their solubility and applications [23–25] has meant that the most important application of IR spectroscopy in this respect is the structural analysis of chemically modified forms of chitin or chitosan. Typical structural analysis of chitin/chitosan derivatives by FTIR spectroscopy involves: (1) FTIR analysis of chitin/chitosan, (2) FTIR analysis of the chemical reagent(s) used in the reaction, (3) FTIR analysis of the chitin/chitosan derivative obtained, (4) identification of differences between the spectra, (5) interpretation of results.

**Figure 10:**
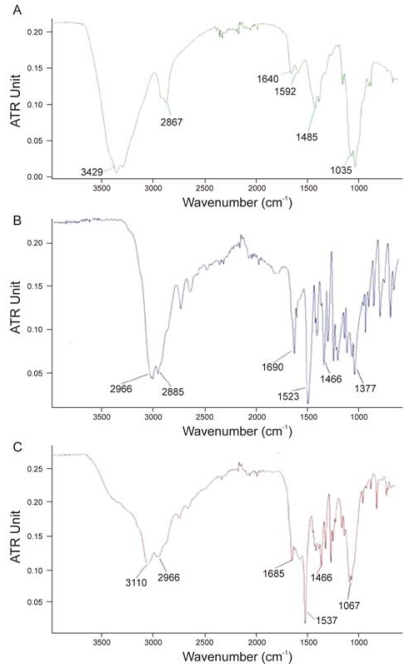
FTIR spectra of (A) chitosan (C), (B) l-glutamic acid (l-GA), and(C) chitosan- l-glutamic acid derivative (Cl-GA). Reprinted from Carbohydrate Polymers 76, 2009, Singh, J., Dutta, P.K., Dutta, J., Hunt, A.J., Macquarrie, D.J., Clark, J.H., Preparation and properties of highly soluble chitosan-l-glutamic acid aerogel derivative, 188–195, Copyright (2010), with permission from Elsevier [144].

An example of the application of FTIR spectroscopy in the structural analysis of chitin and chitosan derivatives is the FTIR analysis of chitosan-l-glutamic acid aerogel derivative, which is soluble over a wide pH range [144]. The FTIR spectra obtained during these investigations are presented in Figure 10.

The following different characteristic bands were assigned from the FTIR spectra of chitosan, l-glutamic acid (l-GA) and chitosan-l-glutamic acid derivative (Cl-GA) (Figure 10):

- ▪ in the IR spectra of chitosan (Figure 10A): 3429 cm^−1^ (O-H stretching overlapping the N-H stretching), 2921 and 2867 cm^−1^ (C-H stretching), 1640 cm^−1^ (amide II band, C-O stretching of the acetyl group), 1592 cm^−1^ (amide II band, N-H stretching) 1485–1380 cm^−1^ (asymmetrical C-H bending of the CH_2_ group) and 1035 cm^−1^ (O bridge stretching) of the glucosamine residue.
- ▪ in the IR spectra of l-GA (Figure 10B): 2966 cm^−1^ (O-H stretching), 2855 cm^−1^ for (C-H stretching), 1690 cm^−1^ (C=O group) and 1523 cm^−1^ (N-H stretching of the amino group),
- ▪ in the IR spectra of Cl-GA derivative (Figure 10C): 3110 and 2966 cm^−1^ (axial OH group of chitosan and glutamic acid), 1685 cm^−1^ (amide linkage), 1556 cm^−1^ (N-H bending and stretching) and 1067 cm^−1^ (C-O-C bridge stretching) of the chitosan residue, 1466 cm^−1^ (the asymmetrical deformation of CH_2_).

The following changes were observed in the IR spectra of the chitosan derivative: the C-O adsorption peak of the secondary hydroxyl group enlarged and moved to 1067 cm^−1^, the intensity of primary alcohol 1035 cm^−1^ (C-O stretching vibration) became much smaller than in chitosan, and a new peak appeared at 2966 cm^−1^, indicating the incorporation of the l-glutamic acid moieties. These FTIR results provided evidence for the formation of an amide linkage between the COOH group of l-GA and the NH_2_ group of the main chitosan chain.

FTIR spectroscopy has been applied to confirm the chemical structure of (1) water-soluble chitosan derivatives such as ethylamine hydroxyethyl chitosan (EHCs) [145], *N*-propyl-*N*-methylene phosphonic chitosan [93], *O*-succinyl-chitosan [146], *N*-alkylated chitosan [147] or methoxy poly(ethylene glycol)-grafted chitosan [148]; (2) organic-soluble derivatives, e.g., acetylated chitosans [149,143]; (3) nanoparticle-chitosan composites [150–152]; 4) hydrogels [153,154] or chitosan-graft copolymers [155–157].

Furthermore, FTIR analyses enabled the interactions occurring between chitin/chitosan and the analysed compounds to be studied and explained [107,158–165]. This technique was applied, for example, in an attempt to obtain an explanation for the sorption mechanism of acid dyes [166–168], phenol and *o*-chlorophenol [169] and fluoride [170,171]. FTIR spectroscopy has also been used to establish differences in structure and the degree of substitution of chitin, chitosan and dibutyrylchitin [172,173].

### 2.3. UV-Vis spectroscopy

Ultraviolet/visible (UV-Vis) spectroscopy is useful as an analytical technique for two reasons. Firstly, it can be used to identify certain functional groups in molecules, and secondly, it can be used for assaying. Unlike IR spectroscopy, UV-Vis spectroscopy involves the absorption of electromagnetic radiation from the 200–800 nm range and the subsequent excitation of electrons to higher energy states. The absorption of ultraviolet/visible light by organic molecules is restricted to certain functional groups (chromophores) that contain valence electrons of low excitation energy. The UV-Vis spectrum is complex and appears as a continuous absorption band because the superimposition of rotational and vibrational transitions on the electronic transitions gives a combination of overlapping lines. Nowadays, the individual detection of electron transfers without superimposition by neighboring vibrational bands can also be recorded [174]. With UV-Vis spectroscopy it is possible to investigate electron transfers between orbitals or bands of atoms, ions and molecules existing in the gaseous, liquid and solid phase. Analysis of solutions and crystals usually takes place in transmission, whereas powdered samples are often measured in diffuse reflection mode (Diffuse Reflectance Spectroscopy–DRS). Unlike IR spectroscopy, where Fourier transform techniques predominate, dispersive spectrometers are almost exclusively used in UV-Vis spectroscopy because of the large band widths. More details about UV-Vis spectroscopy and its application are presented in many papers and books [174–177].

#### 2.3.1. Typical conditions of UV-Vis measurement

UV-Vis spectra of chitin/chitosan derivatives are usually recorded in aqueous acid (acetic acid, phosphoric acid, perchloric acid, hydrochloric acid) solutions in a 1.0 cm quartz cell at ambient temperature [160,178–180]. Sometimes water [181], an aqueous base [182] or DMSO [144] solutions are applied. The Diffuse Reflectance UV-Visible (DRUV) spectra of powdered or film samples are measured [107,112,183–185]. Analysis in the vacuum ultraviolet through the near-infrared range has also been applied [185,186].

#### 2.3.2. UV-Vis spectra of chitin and chitosan

**Figure 11:**
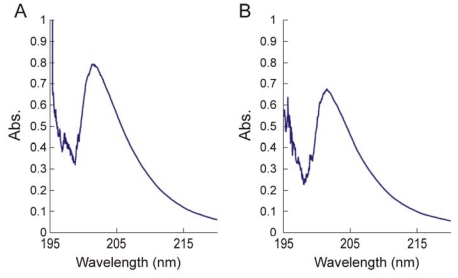
UV spectra: (a) a mixture of *N*-acetyl-glucosamine and glucosamine hydrochloride in 0.1 M hydrochloric acid solution; (b) chitosan in 0.1 M hydrochloric acid solution. Reprinted from Carbohydrate Research 341, 2006, Liu, D., Wei, Y., Yao, P., Jiang, L., Determination of the degree of acetylation of chitosan by UV spectrometry using dual standards, 782–785, Copyright (2010), with permission from Elsevier [180].

Chitin and chitosan include various ratios of two far-UV chromophoric groups, *N*-acetyl-glucosamine (GlcNAc) and glucosamine (GlcN); as a result, their extinction coefficients for wavelengths shorter than approximately 225 nm is non-zero. Because GlcNAc and GlcN residues show no evidence of interacting within the chitin/chitosan chain, the monomer units contribute in a simple, additive way to the total absorbance of these polymers at a particular wavelength [180]. The UV spectra of mixtures of *N*-acetyl-glucosamine and glucosamine hydrochloride are quite similar to the spectra of chitosan, and the λ_max_ is 201 nm in 0.1 M HCl solution (Figure 11) [180].

UV-Vis spectroscopy can be used for the optical characterization of the source of chitin [112]. The optical spectra for two black coral species, *Antipathes caribbeana* and *A. pennacea*, showed a wide band from 300 nm to 500 nm, with small differences depending on the species. Additionally, the spectra confirmed the presence of Fe^+3^ in *A. caribbeana* and Mn^+3^ in both species.

#### 2.3.3. Determination of the degree of *N*-acetylation of chitin and chitosan using UV-Vis spectroscopy

The first derivative UV method for DA determination was proposed by Muzzarelli and Rocchetti in 1985 [187]. The principle of this method was based on the absorbance of the intensity of the *N*-acetyl group in chitin or chitosan. The absorbance of *N*-acetyl glucosamine (at maximum wavelength) was linearly dependent on its concentration in the 0.50–5.0 mg·L^−1^ range. The limit of detection of GlcNAc in 0.01 M acetic acid was found to be 0.5 mg·L^−1^. The DA values were determined in acetic acid solutions at 199 nm [187]. The simplicity and convenience of this method were the best among the available methods, because it required very small amounts of sample and did not require expensive equipment. In addition, the first derivative of the spectra was less affected by background noise and impurities. Several modified first-derivative UV methods have been proposed to improve the convenience and accuracy of measurement [178,180,188–191]. Tan *et al.* [178] compared the results of four methods (^1^H NMR, UV, ninhydrin assay and potentiometric titration) and suggested the first-derivative UV method as a standard method for the routine determination of DA of chitosan. Pedroni *et al.* [188] analysed chitosan solutions in different solvents; 0.1 M HCl solutions turned out to be the best. Hsiao *et al.* [189] suggested concentrated phosphoric acid as the UV-transparent solvent system. In this way, the UV determination could be validated across the whole DA range. Liu *et al.* [180] employed two standards (*N*-acetyl-glucosamine and glucosamine hydrochloride) to determine the degree of *N*-acetylation of chitosan by UV spectrophotometry. Wu and Zivanovic [190] plotted the first derivative UV values at those wavelengths against the concentrations of GlcNAc and found that the best linear regression was obtained at 203 nm (the range of 0–50 μg/mL, R^2^ = 0.996). Da Silva *et al.* [191] put forward a new mathematical expression. This was derived in such a way that DA could be determined directly from the mass concentration of a chitosan solution and the first derivative of its UV spectrum at 202 nm, thus eliminating the need for the empirical correction of curves for highly deacetylated samples. A procedure was proposed for the accurate mass determination of the hygroscopic chitosan.

In summary, UV techniques for DA determination are more sensitive than IR, ^13^C NMR and ^15^N NMR spectroscopy, and they give a better accuracy than NIR, IR and NMR methods [122,192]. The first-derivative UV method is the most sensitive of the proposed techniques. The first derivatives of the spectra are less affected by impurities such as protein, and more accurate data can be obtained. In contrast to other techniques, it is possible to analyse the DA values of a chitin/chitosan sample with a high water content. The first-derivative UV methods are easy to carry out, and the equipment is available in most monitoring and research laboratories. These techniques were used to verify the validity of several methods of DA determination in different laboratories.

#### 2.3.4. Application of UV-Vis spectroscopy to the analysis of chitin/chitosan based compounds

The steps in the structural analysis of chitin/chitosan derivatives by UV-Vis spectroscopy are very similar to those in the FTIR methodology (Section 2.2.4). The main difference is connected with the aim of these analyses. IR spectroscopy is used mostly for determining the molecular structure of chitin and chitosan samples, whereas UV-Vis spectroscopy is more often applied to the study of covalent and non-covalent interactions. Since certain functional groups present in organic molecules absorb light at characteristic wavelengths in the UV-Vis region, this technique is applied qualitatively to identify the presence of these groups in samples, supporting structural information obtained from other spectroscopic methods, especially IR.

**Figure 12:**
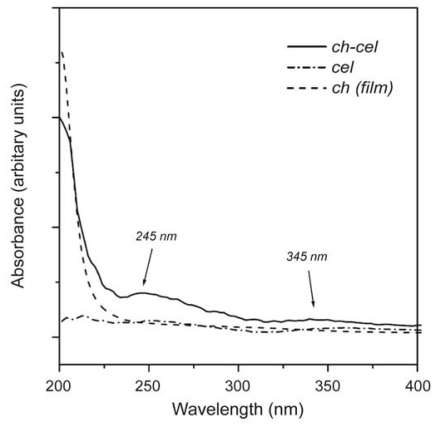
UV–Vis spectrum of a pure chitosan (ch) film measured in transmission mode, and diffuse reflectance UV–Vis spectra of untreated cellulose (cel), and cellulose treated with 1% chitosan (ch–cel). Reprinted from European Polymer Journal 42, 2006, Urreaga, J.M., de la Orden, M.U., Chemical interactions and yellowing in chitosan-treated cellulose, 2606–2616, Copyright (2010), with permission from Elsevier [107].

As mentioned, one of the most important applications of UV-Vis techniques is the characterization of interactions between chitin or chitosan and the target compounds. For example, the analysis of the interaction between chitosan and cellulose, described by Urreaga and de la Orden [107], will be presented in greater detail. The UV-Vis spectra obtained during that study are shown in Figure 12.

The spectrum of chitosan-treated cellulose (Figure 12) showed the characteristic chitosan absorption below 220 nm revealing the presence of chitosan. Additionally, this spectrum showed very weak absorptions at λ > 230 nm, including weak but characteristic bands centred at *circa* 245 nm and 345 nm, which did not appear in the spectra of the starting materials (cellulose and chitin). This observation suggested the existence of chemical interactions between cellulose and chitosan. In order to verify the existence of the above chitosan–cellulose interactions, samples of pure chitosan, pure cellulose and chitosan-coated cellulose were heated at different temperatures and characterized by UV-Vis spectroscopy. The spectrum of the chitosan-treated cellulose was more complex than those of chitin and cellulose and exhibited two new absorptions centred at *circa* 272 and 340 nm. The presence of the band at 272 nm was explained by the independent oxidations of chitosan and cellulose, but the appearance of the 340 nm band again indicated the existence of a chemical reaction in chitosan-treated celluloses, demonstrating that chemical interaction between cellulose and chitosan took place even at moderate temperatures. The new bands, which were observed in materials processed at room temperature, were assigned (also using FTIR and fluorescence spectroscopy) to conjugated imines, produced in the condensation reaction between chitosan amino and carbonyl groups. UV-Vis spectroscopy has also been employed, e.g., to characterize the interactions between chitosan and uranyl ions [160], chitosan and synthetic phospholipid membranes [193], β-cyclodextrin-linked chitosan and p-nitrophenolate [194] and between chitosan and gelatin [195].

UV-Vis techniques were useful for determining the structure of many chitin/chitosan derivatives, for example, chitosan hydrogel membranes obtained by UV- and γ-radiation [196], substituted polyaniline/chitosan composites [163], chitosan-l-glutamic acid aerogel derivatives [144], fluorescent chitosan derivatives containing substituted naphthalimides [197] and dendritic polyaniline nanoparticles synthesized by carboxymethyl chitin templating [103]. They were also employed to determine the actual degree of substitution of fluorescent chitosan (*DS* parameter) and the concentration of the 9-anthrylmethylated unit in modified chitosan [198].

It should also be mentioned that UV-Vis spectroscopy is used to monitor chemical reactions [185,199,200] and the manufacture of complex multilayers, nanoparticles, nanocomposite or multilayer films [183,184,201–203].

UV-Vis spectroscopy is employed to determine the physicochemical properties of chitin/chitosan-based compounds such as the turbidity (optical dispersion, OD) of their solutions [179], the capacity adsorption of chitosan membranes [204], the lower critical solution temperature (LCST) of a chitosan derivative copolymer solution in PBS [205] or the optical properties of nanoparticles [206].

### 2.4. Mass spectrometry

Mass spectrometry (MS) is a destructive analytical technique used for measuring the characteristics of individual molecules. The basic information obtained from mass spectrometric analysis is the molecular mass of a compound, which is determined by measuring the mass to charge ratio (m/z) of its ion. With the ionization method, full particulars about a molecule’s chemical structure can be found. MS can analyse chemicals with a wide mass range–from small molecules to complicated biomolecules such as carbohydrates, proteins, peptides or nucleic acids. The mass spectrometer is composed of three fundamental parts: an ion source, an analyser and a detector. First, the sample must be introduced to the heart of each mass spectrometer–the ion source. There are two possible ways of doing this: through a direct inlet (septum inlet, syringe, probe, *etc.*), or an indirect inlet (gas chromatograph, liquid chromatograph, or capillary electrophoresis column). In the ion source the sample molecules are ionized by one of a variety of methods (electron impact–EI, chemical ionization–CI, electrospray ionization–ESI, matrix assisted laser desorption ionization–MALDI, *etc.*). Next, the stream of ions is transferred to the analyser, where they are sorted and separated according to m/z. The most commonly used analyzers are quadrupoles, ion trap and time of flight. In tandem mass analysis (MS/MS, MS_n_) a combination of two or more analysers is used. The MS can be operated in three modes: full scan (scanning of a selected mass range), more sensitive SIM (selected ion monitoring = SIR - single ion recording), and the most sensitive MRM (multiple reaction monitoring). Finally, the ions reach the detector when their energy is converted to electrical signals that the computer can read. In the end, mass spectra are obtained, and identification of compounds is possible [207–209].

#### 2.4.1. Typical conditions of mass spectrometric analysis of chitin/chitosan and their derivatives

Different types of mass spectrometer can be chosen according to the information required. Spectrometers with various combinations of inlet systems, ion sources and analysers are used for investigations into chitin, chitosan and their derivatives. In most cases the positive ion mode is preferred.

The first method used was pyrolysis-mass spectrometry [210]. That was back in 1982, but combined with gas chromatography it is still a routine technique [211–215]. In GC/MS, the first sample is pyrolyzed (450–615 °C), after which the volatile pyrolysis products are separated on a capillary column (the temperature program from 35–50 °C with different rates to 220–300 °C). In GC/MS analysis, the full scan mode (m/z from 15–35 to 300–650) and the electron impact mode (70 eV) are commonly used. Products are identified on the basis of their GC retention parameters and their mass spectra [210–215].

GLC-MS is used for analyzing chitosan or its impurities. But first, all the target compounds have to be converted to volatile derivatives (hydrolysis, and reduction of chitin [216,217]; this is then followed by the extraction of impurities [218] and trimethylsililation or acetylation). The products are separated using a GLC nonpolar or medium-polar capillary column with a gradient temperature programme (usually from ~60 °C to ~280 °C with different heating rates). For detection, MS with EI ionization is employed [216–218].

Most of the papers dealing with the mass spectrometric analysis of chitin and chitosan report that MALDI was the ionization method selected. This ion source is most often combined with a time of flight analyser (MALDI-TOF) [219–226]. For this type of analysis chitooligosaccharides were dissolved in water [226], MeOH-H_2_O (1:1) [224] or 1% acetic acid solution [225]. The matrix most often used is 2,5-dihydroxybenzoic acid (DHB) [219,221–226]; sometimes it is 2-(4-hydroxyphenylazo)benzoic acid (HABA) dissolved in dioxin [224]. To obtain monosodium and monopotassium carbohydrate adducts in the mass spectrum, NaCl and KCl can be added as a cationating agent [224]. The MS ion source is equipped with a nitrogen laser emitting at 337 nm [225,226]. For these analyses an accelerating potential of 20–30 kV is used, and mass spectra are measured in positive ion mode [221,222]. A modified version of MALDI-TOF was introduced for chitin/chitosan investigations–MALDI-TOF PSD MS (postsource decay mass spectrometry) [227]. Another MS technique with a MALDI source is tandem mass spectrometry, for which an ion trap analyser is very useful. One analyzer allows MS^2^ and also MS^3^ spectra to be obtained using a collision energy previously established experimentally [228]. MALDI-TOF/TOF equipment can also be applied in chitin and chitosan analysis using tandem mass spectrometry. Precursor ions are accelerated at 8 kV, while their fragment ions are accelerated to 19 kV [229].

Time-of-flight secondary ion mass spectrometry (TOF-SIMS) is another method used during chitosan investigations. This type of spectrometer is equipped with a gallium primary ion beam operated at 15 kV. The secondary ions appearing are extracted (10 kV) and fly through the analyzer (TOF type) to the detector [230,231].

After MALDI, ESI (electrospray ionization) is the second most important ionization technique for chitin/chitosan analysis [219,232–238]. The ESI ion source is usually coupled to an ion trap analyser [235], but a quadrupole type of analyser can also be used [237]. For sample measurements several parameters should be optimized, for example, the source voltage (3.5–4 kV), nitrogen sheath gas rate and capillary temperature. A syringe pump is used (3–4 μL/min) for direct sample injection [235,238]. Tandem mass spectrometry with ESI ionization is preferred for acquiring more structural information. If the triple quad system (QqQ) is used, the first quadrupole selects the precursor ions, the second quadrupole acts as collision chamber, and the third quadrupole analyses the ion products; argon is the neutral collision gas [239,240]. Sometimes, a combination of quadrupole and time-of-flight (Q-TOF) is found useful [241]. Connecting a liquid chromatograph to the MS system is an additional advantage. Separating solutions containing chitosan products on a chromatographic column makes the analysis easier. LC-MS analysis is carried out using the same mass spectrometric modes (positive and negative ion detection, electrospray ionization, scanning and SIM mode, *etc.*). Ion source conditions usually are the same as in classic MS analysis that yields protonated products [M+H]^+^, sodium adducts [M+Na]^+^, *etc.* For HPLC separation a different type of column is used, and the mobile phase often consists of acetonitrile or water with added formic acid [242–245]. LC-MS is highly suitable method for investigating chitosan derivatives.

Fast atom bombardment (FAB) is another soft ionization method useful in chitin and chitosan investigations. For this purpose the following compounds can act as the matrix: thioglycerol [246,247], m-nitrobenzyl alcohol [247], glycerol [248–250], dithiothreitol [251], 2,4-dinitrobenzyl alcohol, or dithiothreitol-thioglycerol (1:1, v/v) [223]. The mass spectrometer usually operates in positive-ion mode, most often producing [M+H]^+^ ions [246,248,249,252] but also [M+Na]^+^ [250,251] or [M+K]^+^ [251]. The atom gun is operated at 6–8 kV with xenon as the bombarding gas [246,247,250,251]. This type of ion source cooperates with collision-induced dissociation tandem mass spectrometry (FAB-CID) measurements [246,247,252]. To obtain FAB-CID-MS/MS spectra, the collision is caused by helium, and a 10 kV accelerating voltage is used [246,247].

Sometimes less popular ionization methods are used for chitin/chitosan analysis. One of them is chemical ionization (CI) with the use of ammonia [253] or acetic acid [120] as the reactive gas. In both cases MS works in positive ion mode, generating [M + NH_4_]^+^ and [M + H]^+^, respectively [120,253]. Another ionization method is plasma desorption by the fission products of californium-252 in a time/flow biochemical mass spectrometer [254].

TC-EA/IRMS (high-temperature conversion-elemental analyser/isotope ratio mass spectrometer) is very rarely used in chitin/chitosan investigations. In this method the first step is the quantitative conversion of oxygen to CO and hydrogen to H_2_ at a high temperature. The gaseous products are separated by GC, transferred to IRMS where the ratios of the stable oxygen and hydrogen isotopes in chitin are determined [255,256].

Chitosan TG-MS (thermogravimetry-mass spectrometry) analysis begins with the very rapid heating of the sample from room temperature to 600 °C, after which the gaseous products pass into the mass spectrometer. Ions with m/z in the 40–130 range are analysed by MS with a quadrupole analyzer and Channeltron detector [257].

#### 2.4.2. Mass spectrometric determination of the degree of polymerization of chitin and chitosan

Being a natural co-polymer of glucosamine and *N*-acetyl-glucosamine, chitosan possesses properties like antimicrobial and antitumor activity, and stimulates plant growth [222,238,258]. This activity is closely related to the molecular weight. This fact must be taken into consideration if one is planning to use chitosan in biological or pharmaceutical applications. Chitooligosaccharides can be obtained by depolymerization. This is done physically [226,238] chemically [245] or enzymatically [244,250,259–262]. In each case, MS can be used to determine the degree of depolymerization of chitosan and to track the kinetics.

**Figure 13:**
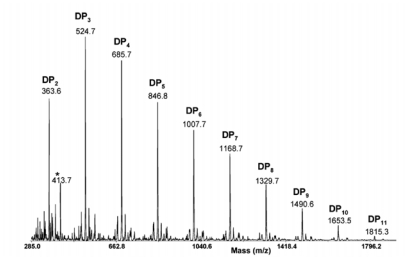
MALDI-TOF MS of deacetylated chitooligosaccharides obtained from ultrasonically treated chitosan. Reprinted with permission from *Biomacromolecules*. **2009**, *10*, 1203–1211. Copyright 2010, American Chemical Society [226].

One of the physical methods for depolymerizing chitosan is sonolysis. It was found that after ultrasound treatment random depolymerization of chitosan occurred, producing many different chitooligosaccharides (COS). ESI-MS [238] and MALDI-TOF MS (Figure 13) [226] were used to detect the products. The primary chemical structure was found to have remained unchanged and that sonolysis was a good enzymatic or alternative chemical method for obtaining oligosaccharides for commercial use.

**Figure 14:**
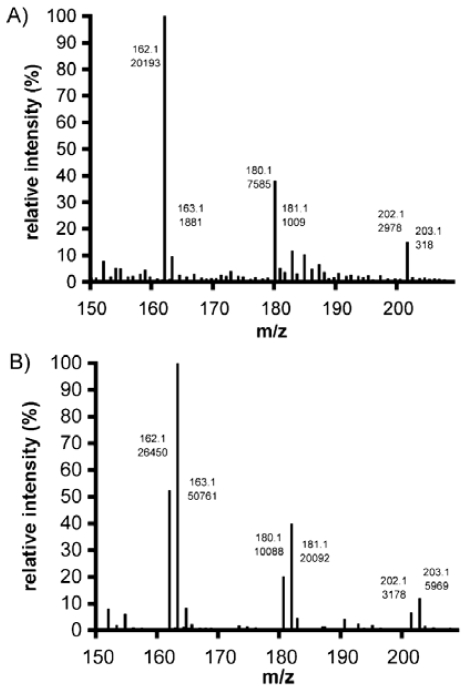
Mass spectra of acid hydrolyzed chitin from *P. chrysogenum* growing on (A) minimal medium, (B) medium with the addition of (^15^NH_4_)_2_SO_4_ (rich medium–Blakeslee’s formula). Reprinted with permission from *Biomacromolecules*. **2009**, *10*, 793–797. Copyright 2010, American Chemical Society [245].

Depolymerization of chitin/chitosan can be achieved by classical chemical methods. The results of LC-MS analysis proved that hydrolysis of chitin from fungi with 6 M HCl yielded glucosamine monomers [245]. Besides providing qualitative chitin composition, the method also makes it possible to track the kinetics of the reaction by ^15^N labeling of the glucosamine from the fungal chitin. For this purpose, acid-hydrolyzed chitin from the fungus *P. chrysogenum* growing on a minimal medium and on a medium containing added (^15^NH_4_)_2_SO_4_ (rich medium–Blakeslee’s formula) was analyzed by LC-MS. Then the analysis on the basis of the peak ratios in mass spectrum was performed (Figure 14) [245].

The enzymatic method of chitin/chitosan depolymerization is the most popular one, and mass spectrometry is often used to detect the products and the efficiency of this process. From MS studies it was found that immobilization of enzymes (papain [259], neutral protease [243]) enhanced depolymerization in the chitosan chain comparing with free enzymes [243,259]. The activities of enzymes isolated from different sources (commercial enzymes [261], isolated from living organisms: *Vibrio harveyi* [244], *Serratia marcensces* [251], *Bacillus circulans* [237], *Amycolatopsis orientalis*, *Streptomyces* sp. [241]) were tested by qualitative MS studies of obtained chitooligosaccharides. It is possible to study enzyme selectivity, and the mechanism of COS bonding to the enzyme. Knowledge of the influence of polymerization and the degree of acetylation is very important in the large-scale production of chitooligosaccharides, and in their use in enzymology or drug design. Table 1 shows a number of examples of the analysis of the degree of polymerization of chitooligosaccharides using mass spectrometry.

To prepare oligosaccharides, chitosan can be hydrolyzed by a mixture of different enzymes (cellulose, alpha amylase, proteinase). The products are analyzed by MALDI-TOF [220].

Mass spectrometry is a good method for the detection of chitooligosaccharides obtained not only by depolymerization, but also as a result of synthesis. FAB-MS and FAB-MS/MS were used to analyse chitinpentaose, chitintetraose, chitintriose and two modified chitin oligosaccharides produced by Rhizobium NodC protein in *Escherichia coli* [246].

#### 2.4.3. Determination of the degree of *N*-acetylation of chitin and chitosan using mass spectrometry

The degree of *N-*acetylation is a factor that allows chitin and chitosan to be distinguished. Besides UV, IR and NMR spectroscopy, mass spectrometry is one of the spectral techniques used to establish the degree of *N-*acetylation of chitin, chitosan and their derivatives [122].

For this purpose, the pyrolysis-gas chromatography-mass spectrometry (EI ionization) combination is used. From the EI mass spectra it is possible to identify volatile compounds obtained from chitin and chitosan. The peaks at m/z 60 and m/z 42 are derived from *N-*acetyl-glucosamine, while the ions m/z 80 and m/z 67 are from glucosamine. DA is established on the basis of the peak ratios 80:60, 67:60, 80:42, 80:125, 94:125, 80:110. These ratios increase with decrease in the degree of acetylation [122,211,212].

**Figure 15:**
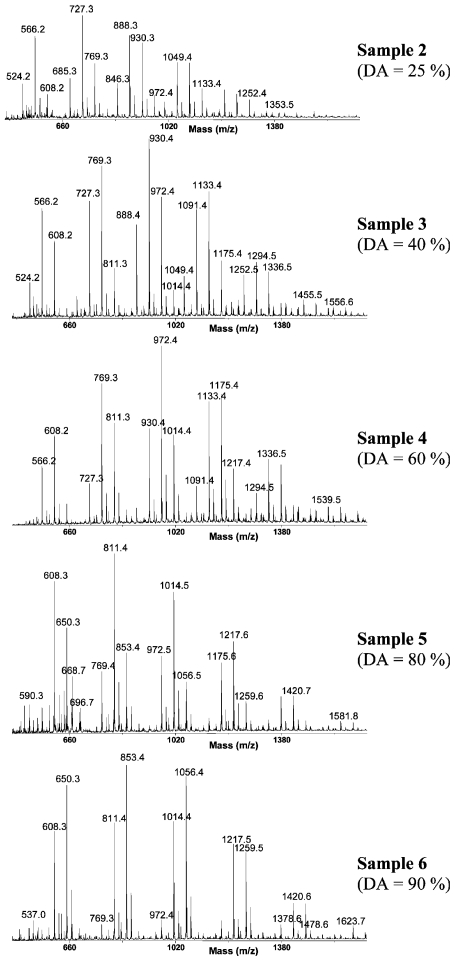
MALDI-TOF MS of chitooligosaccharides with different degrees of acetylation. Reprinted with permission from *Biomacromolecules*. **2008**, *9*, 1731–1738. Copyright 2010, American Chemical Society [240].

As in the case of investigations into the degree of polymerization of chitin/chitosan, the usual MS method is MALDI-TOF. The mass spectra of the series of oligosaccharides with DA from 25 to 90% obtained with this method are shown in Figure 15 [240].

As mentioned above, chitin and chitosan play a significant part in inducing defense responses in plants. Some properties are very selective: for example, chitin oligosaccharides can induce lignifications in wounded wheat leaves, while chitosan COS cannot. Mass spectrometry was used to establish DA in the stereocontrolled synthesis of the chitosan dodecamer (FAB-MS) [263], the synthesis of the chitosan tetramer derivative β-d-GlcNAc-(1→4)- β-d-GlcNAc-(1→4)- β-d-GlcNAc-(1→4)-d-GlcN (FAB-MS) [249], the synthesis of a series of oligosaccharides (MALDI-TOF) [264], and the synthesis of partially *N*-acetylated chitin oligomers using *N*-acylated chitotrioses as substrates in a lysozyme-catalysed transglycosylation reaction (MALDI-TOF, ESI-MS) [219]. All of them are used in biological investigations. The degree of acetylation of enzymatic degradation products of chitin and chitosan is measured. For these investigations chitin deacetylase from *Colletotrichum lindemuthianum* was chosen (FAB-MS, MALDI-TOF) [221,249].

#### 2.4.4. Analysis of chitin and chitosan derivatives using mass spectrometry

Some analytical methods based on mass spectrometry force the conversion of chitin and chitosan into the gaseous state. There are two cases when this is required: during analysis of thermal degradation products, and when GLC-MS is used.

The first example is a study of the thermal decomposition products of chitosan, which is done by TG-MS analysis. The chitosan is heated to different temperatures to be degraded, and the gaseous products are detected using a mass spectrometer. From this experiment it is possible to monitor the thermal behavior of chitosan [257].

In comparison with the previous method, using a combination of pyrolysis-gas chromatographymass spectrometry, it is possible to separate a mixture of chitosan or chitosan pyrolysis products. For qualitative analysis the deuterated analogue of *N-*acetyl-glucosamine is used as an internal standard. The main components of the pyrolysates (chitin markers) are acetamide, acetylpyridone and 3-acetamidofuran [213–215,217]. This method is used to confirm the presence of chitin in biological samples [215,255].

For the GLC-MS analysis of chitin and chitosan, these need to be converted to volatile derivatives. For this purpose the chitosan or chitin methanolysis/hydrolysis products are trimethylsilated or acetylated. The results allow the sugar composition of chitin and chitosan to be determined. For qualitative analysis, an internal standard is needed [216,217].

TC-EA/IRMS is a very interesting, but still rarely used method. It is useful in the palaeolimnological study of fossil chironomid head capsules. All organic compounds are converted to CO and H_2_. With the isotopic ratio mass spectrometer the ratios of stable oxygen and hydrogen isotopes from chironomid larvae can be determined. Water and diet were also investigated in this experiment to test how the δ^18^O and δ^2^H in water and the diet influence the δ^18^O and δ^2^H of chironomid larvae [255,256].

**Figure 16:**
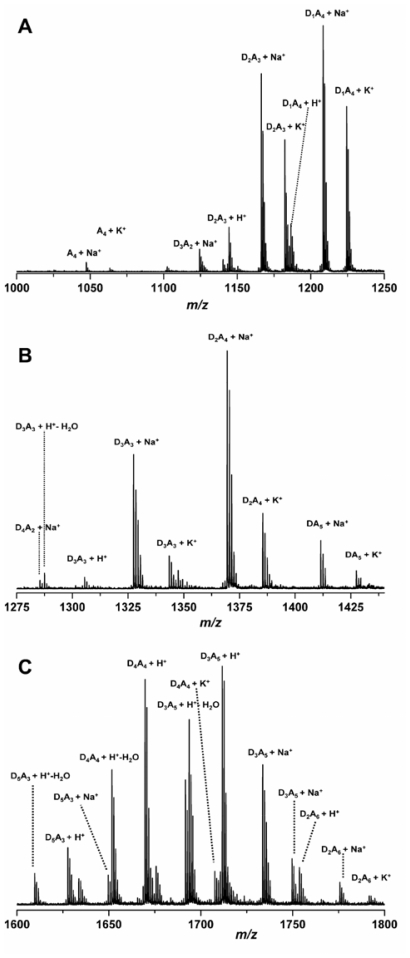
MALDI-TOF-MS of AMAC derivatized chitooligosaccharides: (A) DP 5, (B) DP 6, (C) DP 8. Reprinted from Carbohydrate Polymers 74, 2008, Cederkvist, F.H., Parmer, M.P., Vårum, K.M., Eijsink, V.G.H., Sørlie, M., Inhibition of a family 18 chitinase by chitooligosacharides, 41–49, Copyright (2010), with permission from Elsevier [229].

**Figure 17:**
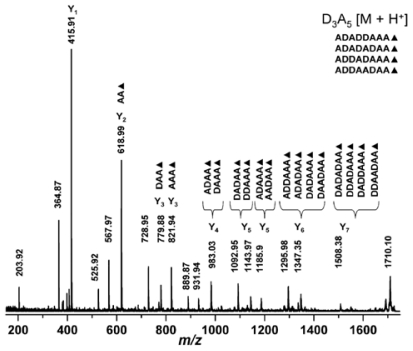
MALDI-TOF/TOF-MS/MS of AMAC derivatized D_3_A_5_ sugar 1711.80 m/z (▴ - AMAC). Reprinted from Carbohydrate Polymers 74, 2008, Cederkvist, F.H., Parmer, M.P., Vårum, K.M., Eijsink, V.G.H., Sørlie, M., Inhibition of a family 18 chitinase by chitooligosacharides, 41–49, Copyright (2010), with permission from Elsevier [229].

Most of all, however, mass spectrometry, especially LC-MS and LC-MS/MS, is used to monitor the success of chitin and chitosan derivatization reactions. The chitosan is derivatized for different reasons, for example, to sequence mixtures of chitooligosaccharides. For this purpose the reducing end of hetero-chitooligosaccharides was treated with 2-aminoacridone (AMAC) by reductive amination. The products were analyzed using MALDI-TOF-MS (Figure 16), MALDI-TOF/TOF-MS/MS (Figure 17) [229], PSD (postsource decay) MALDI TOF MS [227]. Chitosan sequence determination was a part of an investigation into the inhibition of a family of 18 chitinases. Besides AMAC, 3-(acetylamino)-6-aminoacridine is also used for the reductive amination of chitooligosaccharides. The sequencing information was taken from vMALDI (vacuum MALDI) ion trap MS^n^ measurements [228].

Examples of other chitin/chitosan derivatives analysed by MS are listed in Table 2.

#### 2.4.5. Application of chitosan in mass spectrometric analysis

Chitin and chitosan may be involved in an analytical process using mass spectrometry not only as an analyte. These polymers can play an indirect role in MS analysis; given the properties of chitin and chitosan, they can be used in pre-MS analysis treatment.

One example is the coating of capillaries for capillary electrophoresis with carboxymethyl chitosan. In contrast to uncoated capillaries, the results are more stable and repeatable. With this type of capillary for CE-MS, a very good separation efficiency, sensitivity and less interference can be achieved [267,268].

Inductively-coupled plasma-mass spectrometry (ICP-MS) is very commonly applied to determine the trace metals in different matrices like water samples. Chitosan-based chelating resins are the basis of the sample pretreatment method. These were tested for the collection and preconcentration of analytes (metals), for matrix elimination, and to find the best adsorption capacity. These novel resins could be:

- ▪ *N*,*N*′,*N*″-triacetate-type chitosan (EDTriA-type chitosan) [269];
- ▪ Cross-linked chitosan with *N-*2-hydroxypropyl iminodiacetic acid groups (CCS-HP/IDA), and cross-linked chitosan with *N*,*N*- iminodiacetic acid groups (CCS-IDA) [270];
- ▪ Cross-linked chitosan modified with catechol and salicylic acid [271];
- ▪ Chitosan resin derivatized with 3,4-dihydroxybenzoic acid (CCTS-DHBA) [272];
- ▪ Chitosan resin with amino acids [273].

For the same purpose chitosan resin functionalized with histidine was used in a column in combination with an inductively-coupled plasma-atomic emission spectrometer (ICP-AES) [274]. ICP-MS has been used for the meta-analysis of chitosan using samples based on chitosan Chitlacsilver nanoparticles but only to calculate the amount of silver [275].

### 2.5. NMR spectroscopy

NMR spectroscopy is one of the most powerful techniques for the structural and physicochemical study of organic compounds, both small molecules and polymers. It seems to be highly suitable for studying chitin and chitosan. However, the solubility of these compounds presents a serious problem. As stated earlier, chitin with a higher DA is practically insoluble in all solvents and mixtures of different ones, whereas chitosan is soluble in aqueous acidic solution. Its solubility depends on the degree of *N-*acetylation, the degree of polymerization, the degree of crystallinity, the distribution of GlcNAc and GlcN along the polymer chain, the ionic strength of the solvent and the pH and concentration of chitosan in the solution [276].

Different NMR techniques have been used to study chitin, chitosan and their derivatives, including ^13^C [277,278] and ^15^N [279,280] solid-state NMR, and ^1^H [281–283], ^31^P [284], and ^13^C [285] liquid-state NMR. However, only ^13^C and ^15^N solid-state NMR can be used to study chitins with a high DA, as this technique does not require the solubilization of the polymer.

#### 2.5.1. Description of NMR techniques

##### 2.5.1.1. ^1^H NMR spectroscopy

The most important factor is to find a proper solvent, which should have good solubility properties towards the target material. When the sample is only partially soluble, no quantitative analysis is accurate and reproducible [280,286]. Moreover, the residual signal of the solvent should not overlap the signals of the sample. The most common solvents for liquid-state NMR spectroscopy are D_2_O/DCl, D_2_O/CD_3_COOD, D_2_O/DCOOD.

**Figure 18:**
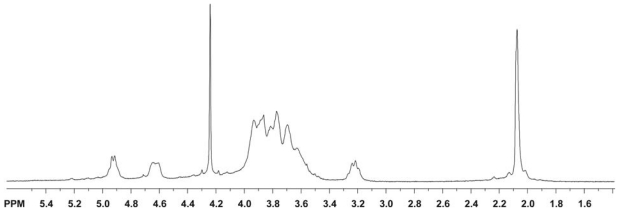
^1^H NMR spectrum of chitosan; the DA = 48. Spectrum was recorded at 400 MHz and 80 °C in D_2_O (pD ~ 5) relative to internal acetone (δ_H_ 2.225).

Before ^1^H NMR measurement, a sample should be freeze-dried 2–3 times from 99.99% D_2_O in order to remove the residual signal of HOD visible in the ^1^H spectrum. ^1^H NMR spectra are usually recorded in aqueous acidic solution (pD ~ 5) at a temperature of 30–80 °C; 8–128 scans are done, depending on the concentration of samples. A typical ^1^H NMR spectrum of chitosan is shown in Figure 18.

The spectrum shows two signals in the anomeric region, namely H-1 of GlcN (D) at δ ~ 4.9 and H-1 of GlcNAc (A) at δ ~ 4.6. They are shifted to higher values due to the neighboring glycosidic and oxygen atoms of the ring. The resonances of H-3–H-6 ring protons of GlcN and H-2–H-6 of GlcNAc are present in the middle of the spectrum (δ ~ 3.5–4.0), forming a group of broad, overlapping signals. The remaining H-2 ring protons of GlcN residues are shifted to characteristically lower values (δ~ 3.2) because of the adjacent amino group. The characteristic signal of the protons in the *N-*acetyl group (GlcNAc) is at δ ~ 2.1.

##### 2.5.1.2. ^13^C NMR spectroscopy

There are two kinds of ^13^C NMR spectroscopy: liquid-state and solid-state. The first one has the same limitations as ^1^H NMR spectroscopy, namely, the insufficient solubility of most analyzed materials. Moreover, ^13^C NMR spectroscopy is much less sensitive than ^1^H NMR spectroscopy owing to the properties of the carbon nucleus and the only 1% abundance of the ^13^C isotope in nature. Liquid-state ^13^C NMR spectra are usually recorded from the same kinds of solutions as are used for producing ^1^H NMR spectra, but the number of scans has to be much higher–this can vary from several dozen to hundreds of thousands. Solid-state ^13^C NMR spectroscopy is a much more powerful technique. Chitin can be analysed without any special solubilization of the polymer. This means that quite a large amount of sample can be used, which solves the problem of the low sensitivity of ^13^C NMR spectroscopy. The ^13^C NMR spectra of solid samples are generally recorded with magic-angle spinning (MAS) and cross-polarization (CP). MAS averages out dipolar interactions and chemical shift anisotropy, producing highly resolved spectra. CP considerably increases the sensitivity of the technique by reducing the relaxation delay due to the magnetization transfer from the ^1^H to the ^13^C spins. The intensities of the ^13^C NMR signals are influenced by the kinetics of the CP process: different contact times affect the intensities of ^13^C NMR resonances. Hence, it is very important to use the proper contact and relaxation delay times [287–289]. Duarte *et al.* optimized the relaxation delay (5 s) and the contact time (1 ms) during series assignments of the degree of *N-*acetylation of chitin and deacetylated chitin samples. Care should be taken to ensure that the CP build-up is similar for all the carbon atoms used in the quantitative calculation. Moreover, different chitin/chitosan materials often contain paramagnetic centres, which may distort the intensity of some ^13^C NMR signals in the spectrum [287].

**Figure 19:**
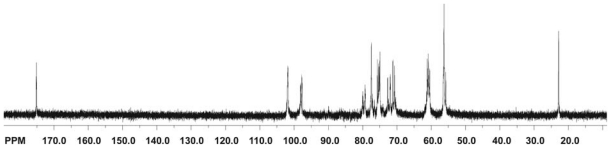
Liquid-state ^13^C NMR spectrum of chitosan; DA = 48. Spectrum was recorded at 100 MHz and 70 °C in D_2_O (pD ~ 5) relative to internal acetone (δ_C_ 31.45).

A typical ^13^C NMR spectrum (Figure 19) of chitin/chitosan contains anomeric (C-1) signals of both monosaccharide residues (GlcN and GlcNAc) at δ ~ 102–105, C-3 and C-5 signals at δ ~ 73–75, C-6 signals at δ ~ 60, C-4 signals shifted to 81–85 due to glycosylation at position 4, and C-2 resonances shifted to δ ~ 55–57 due to the influence of the attached amino group. The remaining signals of the *N*-Ac group of GlcNAc are at δ ~ 23 and δ ~ 174 (methyl group and carboxylic carbon atoms respectively).

The sets of ^13^C chemical shifts for chitin samples isolated from different sources were found elsewhere [53].

##### 2.5.1.3. ^15^N NMR spectroscopy

Solid-state ^15^N NMR spectroscopy, like solid-state ^13^C NMR spectroscopy, is a very useful technique, especially for studying insoluble chitin, but it is more time-consuming owing to the low natural abundance of the ^15^N nuclide (<0.4%). Even using high-field NMR spectrometers with large amounts of sample, measurement times are usually of the order of 12–24 h, which makes the technique very expensive. Watson *et al.* developed the ^15^N labeling of fungal chitin, which makes ^15^N NMR spectroscopy much more effective [245]. The solid-state ^15^N NMR spectra for the natural abundance of ^15^N and for ^15^N-labeled chitin samples needed 60,000 and only 2,000 repetitions respectively. As with solid-state ^13^C NMR spectroscopy, care should be taken to ensure that CP build-up is similar for both *N-*acetylated and *N-*deacetylated nitrogen centres.

Two different nitrogen atom signals are present in the chitin/chitosan spectrum: one corresponds to the amine group (NH_2_ in GlcN; δ ~ 10), the other to the acetamide group (NH-CO-CH_3_ in GlcN; δ ~ 110).

##### 2.5.1.4. ^31^P NMR spectroscopy

^31^P NMR spectroscopy is one of the more routine NMR techniques because ^31^P has an isotopic abundance of 100% and a relatively high magnetogyric ratio. Neither native chitins nor synthesized chitosans contain phosphorus atoms, so they cannot be analysed directly using solid-state ^31^P NMR spectroscopy. However, it is possible to characterize numerous soluble chitosan derivatives containing phosphorus atoms using both liquid-state and solid-state ^31^P NMR spectroscopy. Particular types of phosphorus compounds have characteristic chemical shift ranges and are very easily identified in the spectrum.

##### 2.5.1.5. Two-dimensional (2D) NMR spectroscopy

2D NMR spectroscopy provides much more information about the molecule than 1D NMR and is very useful for studying complex molecules. Of the many 2D NMR spectroscopic techniques, COSY (DQF-COSY), NOESY and HSQC are the most common.

COSY (COrrelation SpectroscopY) and DQF-COSY (Double Quantum Filtered COrrelation SpectroscopY) allow one to determine which protons are spin-spin coupled. The signals of protons that are two or three bonds apart are visible. NOESY (Nuclear Overhauser Effect SpectroscopY) shows correlations of all protons which are close enough for dipolar interaction by coupling through space (<5 Å). ^1^H-^13^C HSQC (Heteronuclear Single Quantum Coherence) allows one to determine which protons are directly bonded with particular carbon atoms.

#### 2.5.2. Determination of the degree of acetylation (DA)

As already stated, determination of DA of chitin and chitosan is very important from the applicative point of view. Apart from UV/Vis and FTIR, NMR spectroscopy is the most powerful technique for such a study. Review articles summarizing the literature to date on the DA determination of chitin and chitosan using NMR methods have been published by Kasaai [122,131,276]. The DA values of chitin and chitosan can be determined on the basis of integrals from different NMR spectra, *i.e.*, liquid state ^1^H, and ^13^C (spectra decoupled from ^1^H during the acquisition time, without the NOE effect) [290], and solid-state ^13^C and ^15^N. For instance, for ^1^H and ^13^C NMR spectra, the DA can be calculated by comparing the integral of the methyl carbon/protons of the acetyl group to the integrals of other carbons/protons from the main chains. Many equations for the DA calculation have been put forward for different types of NMR spectroscopy. DA of chitosan can be calculated on the basis of proton integrals from ^1^H NMR spectra using several equations:

(4) $$\%DA=\frac{1/3*I_{{CH}_{3}(A)}}{1/6*I_{H2-H6(A+D)}}*100$$

[178,291]

(5) $$\%DA=\frac{7(I_{H1(A)}+I_{{CH}_{3}(A)})}{4(I_{H1(D)}+I_{H2-H6(A+D)})+I_{H1(A)}+I_{{CH}_{3}(A)}}*100$$

[282]

(6) $$\%DA=\frac{I_{{CH}_{3}(A)}}{3*I_{H1(A+D)}}*100$$

[290]

(7) $$\%DA=\frac{I_{H1(A)}+I_{{CH}_{3}(A)}/3}{I_{H1(D)}+I_{H2(D)}+I_{H1(A)}+I_{{CH}_{3}(A)}/3}*100$$

[212]

(8) $$\%DA=\frac{1/3*I_{{CH}_{3}(A)}}{I_{H2(D)}}*100$$

[292]

(9) $$\%DA=100-\frac{I_{H1(D)}}{I_{H1(D)}+I_{{CH}_{3}(A)}/3}*100$$

[293]

(10) $$\%DA=100-\frac{I_{H1(D)}}{I_{H1(D)}+I_{H1(A)}}*100$$

[293]

and the averaged value of three equations [128]:

(11) $$\%DA=100-\frac{I_{H2(A)}+I_{H3-H6(A+D)}}{I_{H2(D)}}*100$$

(12) $$\%DA=100-\frac{I_{H2(A)}+I_{H3-H6(A+D)}}{I_{{CH}_{3}(A)}}*100$$

(13) $$\%DA=100-\frac{I_{H2(D)}}{I_{{CH}_{3}(A)}}*100$$

On the basis of carbon atom integrals from ^13^C NMR spectra, DA of chitosan is calculated mainly according to equation [130,278,288,289,294]:

(14) $$\%DA=\frac{I_{{CH}_{3}(A)}}{I_{C1-C6(A+D)}/6}*100$$

Since solid-state ^15^N NMR spectra of chitin and chitosan show two very well resolved nitrogen atom signals, DA can be calculated using a simplified equation [279,280]:

(15) $$\%DA=\frac{I_{N(A)}}{I_{N(A)}+I_{N(D)}}*100$$

There are many reports on the use of different NMR spectroscopy methods for determining DA of chitin and chitosan. In 1990, Hirai *et al.* suggested using ^1^H NMR to study the degree of acetylation of chitosan with different DA (3–40%) [291]. The proposed method was more effective, precise and simple compared to the conventional colloid titration and elemental analysis methods, which were also applied during the study. One of the first assignments of the ^1^H and ^13^C chemical shifts of chitosan using ^1^H, ^13^C, and COSY NMR spectra was also reported [291]. In 1990, Pelletier *et al.* used solid-state ^13^C NMR to determine DA of chitin and chitosan products [295]. The percentage of deacetylation was calculated by comparing the area of CH_3_ resonance to the resonances of the all the remaining carbons. Raymond *et al.* used the same approach [278] to compare DA of chitosan obtained by ^13^C CP-MAS NMR spectroscopy and conductometric titration. Although both methods yielded similarly good results, the authors concluded that NMR was somewhat limited when DA was low, and likewise conductometric titration when DA values were high [278]. Ottøy *et al.* investigated chitosans fractionated into acid-soluble and acid-insoluble fractions after heterogeneous alkaline deacetylation [294] and obtained the DA values for both groups of fractions using ^13^C CP-MAS NMR spectroscopy. Comparison of these results with those from ^1^H NMR spectroscopy for the acid-soluble fractions showed them to be consistent.

One of the first applications of solid-state ^15^N NMR spectroscopy to the study of chitin and chitosan was described by Yu *et al.* [279]. The authors used this technique to determine the degree of *N-*acetylation of chitin and chitosan, finding that solid state ^15^N NMR was the more reliable method, especially for the determination of the extent to which poly(3-hydroxybutyrate) was grafted onto chitosan; the drawback of this method is that it is more time-consuming owing to the low natural abundance of ^15^N. These authors noted that ^15^N NMR spectra were easier to analyse than ^13^C CP-MAS spectra, in which all the oxygen-bearing carbons of both chitin and chitosan overlap; this makes calculating DA more difficult [279].

**Figure 20:**
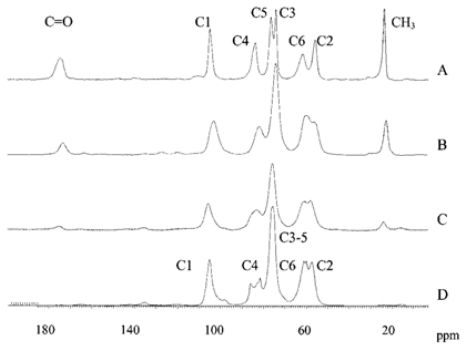
^13^C CP-MAS NMR spectra of samples A-D (with decreasing DA; ~100%, ~60%, ~20%, ~0%). Reprinted with permission from *Biomacromolecules*. **2008**, *1*, 746–751. Copyright 2010, American Chemical Society [280].

**Figure 21:**
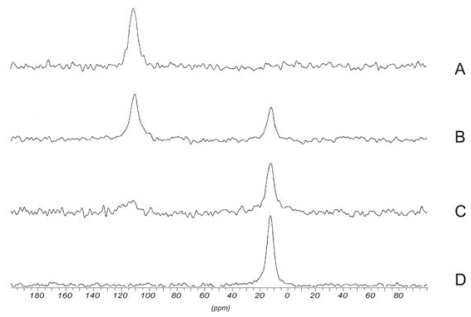
^15^N CP-MAS NMR spectra of samples A-D (with decreasing DA; ~100%, ~60%, ~20%, ~0%). Reprinted with permission from Biomacromol. 2000, 4, 746–751. Copyright 2010, ACS Publications [280].

In 2000 Heux *et al.* compared DA calculated from ^1^H liquid-state NMR, and ^13^C and ^15^N solid-state NMR in the whole range of acetyl content from 0 to 100% [280]. The authors found that all three methods were in good agreement; nevertheless, the limitation of solid-state NMR was reliable detection at DA < 5% due to a small distortion of the spectrum baseline and signal broadening. They also found that ^15^N CP-MAS NMR spectroscopy was particularly powerful for calculating the acetyl content in complex associations of chitin and other polysaccharides [280]. Sets of ^13^C and ^15^N CP-MAS NMR spectra are illustrated in Figures 20 and 21, respectively. The spectra were recorded using the following parameters: for the ^13^C spectra contact time = 1 ms, recycle delay = 4 s, and 10,000 scans were acquired; for the ^15^N spectra the corresponding values were 2 ms, 1 s and 100,000 scans [280].

Detailed descriptions of the determination of DA of chitin or chitosan using ^1^H NMR spectroscopy [128,178,212,282,290,292,293,296], and ^13^C CP-MAS NMR spectroscopy [130, 287–289,297] can be found elsewhere.

It should be mentioned that ^1^H NMR spectroscopy is the best choice for determining DA of chitosan. ^1^H NMR does not require previous calibrations and enables the accurate determination of even low DA values. Moreover, the importance of this method in the study of chitosan is also demonstrated by the fact that ^1^H NMR data are usually used as standards for calibrating alternative methods [296]. Lavertu *et al.* validated the ^1^H NMR spectroscopic method for the determination of DA using equations 1, 6a, and 6b. DA values calculated using three different combinations of peak intensities were very close to each other, demonstrating that the technique is also internally consistent. Moreover, the authors discussed the precision, ruggedness, robustness, specificity, stability and accuracy of the technique [293]. It was found to be simple, rapid and more precise than other known techniques like IR or titration.

#### 2.5.3. Determination of the pattern of *N*-acetylation

**Figure 22:**
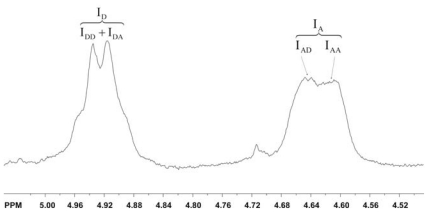
Section of the anomeric region of the ^1^H NMR spectrum of chitosan; DA = 48. Spectrum was recorded at 400 MHz and 80 °C in D_2_O (pD ~ 5) relative to internal acetone (δ_H_ 2.225). Appropriate intensities of sequences are labeled.

Most properties of chitin and chitosan and further applications depend strongly not only on DA, but probably also on the pattern of acetylation (PA) [2]. PA is a parameter describing the distribution of GlcNAc and GlcN residues along the polysaccharide chain and can be determined by ^1^H and ^13^C NMR spectroscopy using appropriate integrals. In the ^1^H NMR spectrum, the nearest neighbor effect on resonances is visible for the anomeric proton of GlcNAc (sequences AD and AA) and proton H-1 of GlcN (sequences DD and DA) (Figure 22). The rest of the signals in the ^1^H NMR spectrum are unsuitable for PA assignment as they overlap [282].

**Figure 23:**
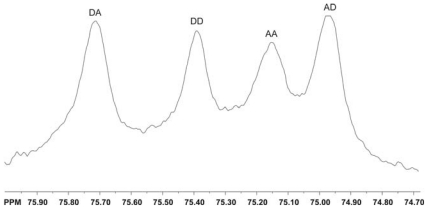
Sections of the ^13^C NMR spectra of chitosan; DA = 48, showing the C-5 signals region. Spectrum was recorded at 100 MHz and 70 °C in D_2_O (pD ~ 5) relative to internal acetone (δ_C_ 31.45). Appropriate sequences are labeled.

In general, PA in the chitosan samples can be calculated from the C-3, C-5 and C-6 carbon signals in the ^13^C NMR [297], although detailed inspection of ^13^C NMR spectra showed that the highest resolution with only low overlapping signals for the four diad frequencies (*F_AA_*, *F_AD_*, *F_DA_* and *F_DD_*) were achieved for the carbon C-5 signals [285] (Figure 23). For the other carbon resonances diad, triad and tetrad peaks were superimposed on neighboring peaks, which precludes a quantitative extraction of signal areas.

The relative experimental intensities of ^1^H or ^13^C resonances in chitosan samples should be normalized according to Bernoullian statistics [298,299] and can be presented as:

(16) $$F_{AD}=\frac{I_{AD}+I_{DA}}{I_{AD}+I_{DA}+I_{DD}+I_{AA}}$$

(17) $$F_{AA}=\frac{I_{AA}}{I_{AD}+I_{DA}+I_{DD}+I_{AA}}$$

(18) $$F_{DD}=\frac{I_{DD}}{I_{AD}+I_{DA}+I_{DD}+I_{AA}}$$

where *I_AD_*, *I_D_*_D_, *I_A_*_A_ and *I_DA_* are the respective intensities of signals AA, DD, AA and DA in the samples. In the above equation *F_AA_* (*F_DD_*) is the probability that two A(D) groups are adjacent to each other and *F_AD_* the probability that one group A has D as a neighbor, and *vice versa*. The data should be transformed into one parameter PA, which describes the acetylation pattern as described before [298,299]:

(19) $$PA=\frac{F_{AD}}{(2F_{AA})+F_{AD}}+\frac{F_{AD}}{(2F_{DD})+F_{AD}}$$

**Figure 24:**
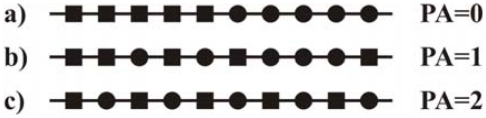
The distribution of GlcNAc (▪) and GlcN (●) residues along the polysaccharide chain at DA = 0.5; (a)–perfect block (PA = 0), (b)–random distribution (PA = 1), and (c)–alternating distribution (PA = 2).

If the statistics are consistent with the Bernoullian model for polymers, the values PA = 0, 1 and 2 respectively indicate a perfect block, random distribution, and the alternating distribution of *N-*acetyl groups along the chitin/chitosan chain (Figure 24).

Determination of PA in a chitosan sample using NMR data requires spectra with sufficient resolution of the diad frequencies. In order to get well resolved spectra, the raw chitosan should be depolymerized by nitrous acid. Additionally, pD ~ 4 and higher measurement temperatures (60–80 °C) respectively improve the solubility of chitosan and the reduce viscosity of the solution.

Vårum *et al.* have reported methods for determining the nearest-neighbor (diad) frequencies *F_AA_*, *F_AD_*, *F_DA_*, and *F_DD_* of chitosans using ^1^H and ^13^C NMR spectroscopy [282,297]. In the first paper, DA and PA of different chitosans obtained by the depolymerization of chitin under homogeneous and heterogeneous conditions were determined by ^1^H NMR spectroscopy. Chitosan prepared by *N-*deacetylation under homogeneous conditions gave values for the diad frequencies that were roughly consistent with a random distribution of the *N-*acetyl groups. Samples prepared under heterogeneous conditions had a frequency of the AA diad slightly higher than that for a random (Bernoullian) distribution. This means that the GlcNAc units of chitosans prepared by *N-*deacetylation under heterogeneous conditions had a slightly more blockwise distribution than those prepared under homogeneous ones [282]. ^13^C NMR spectroscopy results revealed that both groups of chitosans (*N-*deacetylated under homogeneous and heterogeneous conditions) gave values for the diad and triad frequencies that were consistent with a random arrangement of GlcN and GlcNAc residues along the chitosan chain [297]. Kumirska *et al.* compared the most frequently used methods for the PA determination of different chitosan samples [300]. These authors proved that ^13^C NMR spectroscopy based on the analysis of carbon C-5 signals was clearly the most suitable method for determining PA. A number of validation parameters of that method were presented (specificity, sensitivity, repeatability and reproducibility) in that paper [300].

Weinhold *et al.* improved the chitosan pattern determination proposed by Vårum *et al.* [297] through the implementation of a line-fitting procedure for ^13^C NMR spectra [285]. The authors assigned PA (0.5–1.5) to 32 chitosans and did not find any evidence for the existence of a clear blockwise or clear alternating chitosan preparation, although the various samples were produced under different conditions. Moreover, it was shown for the first time that PA was exponentially correlated with DA:

(20) $$PA=1.11-0.58*e^{\left(\frac{-DA}{0.13}\right)}$$

Kumirska *et al.* applied a similar approach for the determination of PA of low-molecular-weight chitosans used in biomedical applications in order to assess the need for the initial depolymerization of the samples [301]. PA values of low-molecular-weight chitosan preparations (<41 kg/mol) were estimated without initial degradation of the native samples, whereas for higher-molecular-weight samples (>41 kg/mol) limited degradation was recommended.

Martinou *et al.* implemented ^1^H and ^13^C NMR spectroscopy to monitor the mode of action of chitin deacetylase from *Mucor rouxii* on fully water-soluble, partially *N-*acetylated chitosans [302]. The chitosan was enzymatically deacetylated and the nearest-neighbor frequencies (*F_AA_*, *F_AD_*, *F_DA_* and *F_DD_*) were determined by ^13^C NMR spectroscopy, showing that the transition frequencies *F_AD_* and *F_DA_* were lower than expected from a random (Bernoullian) distribution in the further enzymatically deacetylated chitosans, while *F_AA_* and *F_DD_* were higher compared to the random distribution. Such a change in the frequencies associated with the enzymatic deacetylation of chitosans was qualitatively in agreement with an enzyme operating according to a multiple-attack mechanism (chitin deacetylase did not preferentially attack any sequence in the chitosan molecule) [302].

#### 2.5.4. Study of chitin and chitosan derivatives

**Figure 25:**
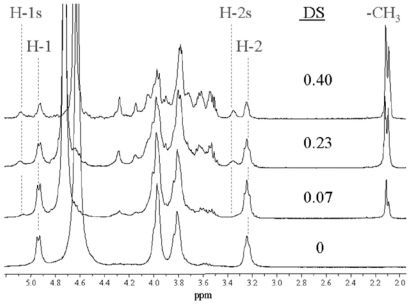
^1^H NMR spectra of chitosan (DP = 25, DA < 0.001) *N-*alkylated with the *N-*acetylated trimer A–A–M. Reprinted from Carbohydrate Research 337, 2002, Tømmeraas, K., Köping-Höggård, M., Vårum, K.M., Christensen B.E., Artursson, P., Smidsrød, O., Preparation and characterization of chitosans with oligosacharide branches, 2455–2462, Copyright (2010), with permission from Elsevier [303].

Chitin and chitosan derivatives can be characterized by all of the above-mentioned NMR techniques in both the liquid and solid states. Very often ^1^H and ^13^C NMR spectroscopy are used together to study soluble compounds, and when phosphorus derivatives are involved ^31^P NMR spectroscopy is additionally invoked. For instance, ^1^H NMR spectroscopy can be used to assign the degree of substitution (DS) of chitosan by modifying groups. Tommerass *et al.* studied the *N-*alkylated trimer 2- acetamido-2-deoxy-d-glucopyranosyl-β-(1→4)-2-acetamido-2-deoxy-d-glucopyranosyl-β-(1→4)-2,5- anhydro-d-mannofuranose (A–A–M) on a fully *N-*deacetylated chitosan using different NMR techniques. The authors used ^1^H NMR spectroscopy to assign the degree of substitution of chitosan (the DA<0.001) by the A-A-M group [303]. The DS values (0.07, 0.23, 0.40) were calculated from the methyl resonance of the *N-*acetyl group at δ 2.1 compared to H-2 of substituted and unsubstituted chitosan residues at δ 3.0–3.4 (Figure 25).

^1^H NMR spectroscopy was used to characterize a number of chitin and chitosan derivatives, *inter alia*, glycol chitosan [304], hydroxypropyl chitosan [155], *N-*(4-bromonaphthalimide)-chitosan [197], acylated chitosan [143,173], *N-*alkylated chitosan [147], and various *N-*alkyl and *N-*benzyl chitosans [305]. ^13^C NMR spectroscopy was applied to study many soluble derivatives of chitin and chitosan: 6-carboxychitin [306], 6-*O*-carboxymethyl-chitin and 3,6-*O*-carboxymethyl-chitin [307], and C-6 and C-3 sulphated chitosan [308]. ^1^H and ^13^C NMR spectroscopy were utilised to characterize *N*,*N*,*N*-trimethyl chitosan chloride [309], 2-*N-*(2-ethoxycarbonylethyl)chitin [310], *O*-succinyl-chitosan [146], chitin-graft-poly(ε-caprolactone) [311], *N-*carboxybutylchitosan and 5-methylpyrrolidinone chitosan [312], and chitosan derivatives with galactose groups [313]. ^1^H and ^13^C NMR spectroscopy were supplemented by DQF-COSY NMR spectroscopy for the study of the *N-*alkylated trimer 2-acetamido-2-deoxy-d-glucopyranosyl-β-(1→4)-2-acetamido-2-deoxy-d-glucopyranosyl- β-(1→4)-2,5-anhydro-d-mannofuranose on a fully *N*-deacetylated chitosan [303], and 2D ^1^H,^13^C HSQC NMR spectroscopy for chitins sulphated at C-3 and C-6 [314], as well as COSY and ^1^H,^13^C HETCOR (hetero-correlation spectroscopy) for the study of *O*,*N*-carboxymethylchitosans [281].

Solid-state ^13^C CP-MAS NMR spectroscopy was used to characterize *O*-hydroxypropyl chitin [315], chitin grafted with poly(acrylic acid) [316], derivatives of chitosan and crown ethers [317], dibutyrylchitin [172], and gels obtained by reacting chitosan with diethyl squarate and 1,1,3,3-tetramethoxypropane [318,319].

^31^P NMR spectroscopy in combination with other NMR techniques was used in the study of numerous chitin and chitosan derivatives containing phosphorus atoms: *N-*(diisopropylphosphono-thiooxomethyl) chitosan, *N-*(2-diethylphosphono-ethyl)-chitosan, and *N-*(2,2-diethylbisphosphono-ethyl)- chitosan [320], phosphorylated chitosan and chitin [321–323], phosphorylcholine chitosan [324], *N-*propyl-*N-*methylene phosphonic chitosan [93], 6-deoxy(diethyl phosphate)-chitin, [325], *O*-ethyl phosphonate chitosan [326], and diethyl phosphate chitosan [327].

#### 2.5.5. Physicochemical characterization of chitin and chitosan

Tanner *et al.* [328] were among the first to attempt to analyze α- and β-chitin polymorphs by NMR spectroscopy. Spectra of different α- and β-chitin were identified, but broad lines and distinct peak asymmetry made it difficult to interpret the spectra, to measure accurate values of chemical shifts, and to make useful comparisons between the spectra of those two polymorphs. In 2004 Jang *et al.* recorded solid-state ^13^C CP–MAS NMR spectra for α-chitin, β-chitin, and γ-chitin [52]. The NMR spectra of α-chitin and β-chitin were clearly distinguished, but the γ-chitin spectrum was almost the same as that of α-chitin. In the β-chitin spectrum, a signal assigned to C3 and C5 appeared at δ 74, whereas in the α-chitin one, the C3 and C5 appeared as two sharply resolved signals at δ 73 and δ 75, respectively. This was attributed to the different configurations of C3 and C5 resulting from the hydrogen bonds formed. α-Chitin, having an antiparallel structure, has intersheet and intrasheet hydrogen bonding, but β-chitin, having a parallel structure, forms only intrasheet hydrogen bonding. A detailed explanation of the two kinds of hydrogen bonding in α-chitin using ^13^C CP-MAS NMR spectroscopy was published by Kameda *et al.* [329]. The peaks corresponding to the C3 and C5 carbon atoms of γ-chitin were resolved at δ 73 and δ 75 because γ-chitin formed intersheet hydrogen bonding [52].

In a similar way, Cárdenas *et al.* characterized different polymorphs of chitins isolated from squid, shrimp, prawn, lobsters and king crabs [53].

Using ^13^C CP-MAS NMR spectroscopy Cortizo *et al.* identified β-chitin isolated from the squid pen *Illex argentinus* [330], and Manni *et al.* isolated α-chitin from shrimp waste [331].

#### 2.5.6. Other applications of NMR techniques

NMR techniques can also be used to examine different chemical and physiochemical processes by the analysis of final or intermediate products. Toffey *et al.* [332] used solid-state ^13^C-NMR spectroscopy to investigate the thermally-induced conversion into chitin of a water-soluble solid consisting of an ionic complex of chitosan, acetic acid and chitosonium acetate. They recorded the ratio of the signal at δ 179 (the carboxylic atom of the acetate group) and the signal at δ 173 (the carboxylic atom of the *N-*acetyl group) during the conversion.

Holme *et al.* studied the thermal depolymerization of chitosan chloride in the solid state by analyzing the products using ^1^H and ^13^NMR spectroscopy [333]. The rate of acid hydrolysis of the glycosidic bond in chitosan solutions was found to be in the order A–A ≈ A–D >> D–A ≈ D–D.

Einbu and Vårum reported studies of the depolymerization and de-*N-*acetylation reaction using the chitin dimer (GlcNAc-GlcNAc) and the monomer GlcNAc as respective model substances. The rates of the reactions were determined as a function of acid concentration and temperature using ^1^H NMR data [334]. In a subsequent paper, the authors described their study of chitin hydrolysis by concentrated and deuterated hydrochloric acid, which was a very suitable solvent for the direct characterization of the chemical composition of chitin by ^1^H NMR spectroscopy. The rate of hydrolysis of a glycosidic linkage following an *N-*acetylated unit was found to be 54-times higher than the rate of *N-*deacetylation and 115-times higher than the rate of hydrolysis of a glycosidic linkage following a *N-*deacetylated unit [335].

NMR spectroscopy can be utilized to study the enzymatic degradation of chitin. Martinou *et al.* used ^1^H and ^13^C NMR spectroscopy to monitor the mode of action of chitin deacetylase from *Mucor rouxii* [302], while Sorbotten *et al.* studied the degradation products of chitosans using chitinase β from *Serratia marcescens* by ^1^H NMR [336].

NMR spectroscopy, molecular modeling (MM) and molecular dynamics (MD) are excellent tools for studying interactions between chitin/chitosan and proteins or peptides. Colombo *et al.* used molecular dynamics (MD) simulations based on NOE NMR to study the complex between hevein and *N*,*N*′-diacetylchitobiose and *N*,*N*′,*N*″-triacetylchitotriose. The results of the simulations showed that a carbohydrate oligomer was able to move on the surface of the relatively flat binding pocket of hevein, therefore occupying different binding subpockets [337]. NMR spectroscopy and MD were also used for studying the interaction of a variety of modified hevein domains with chitooligosaccharides [338]. Aboitiz *et al.* utilized NMR and MM to demonstrate that trisaccharides containing GalNAc and ManNAc residues were also recognized by hevein domains. Their observations indicated that the present nature of the modifications of chitotriose at either the non-reducing end (GalNAc instead of GlcNAc) or at the reducing end (ManNAc instead of GlcNAc) did not modify the mode of binding of the trisaccharide to hevein [339].

### 2.6. Other spectroscopic methods

Apart from the spectroscopic techniques commonly used in the structural analysis of chitin, chitosan and their derivatives, which have been described above, there are many other spectroscopic methods applied to the analysis of these polymers. They include Scanning Electron Spectroscopy (SEM), Scanning Electron Spectroscopy coupled with Energy Dispersive Spectroscopy (EDAX), Scanning Electron Spectroscopy coupled with Transmission Electron Microscope (SEM-TEM system), Circular Dichroism Spectroscopy (CD) and Inductively Coupled Plasma Spectroscopy (ICP).

SEM is an extremely useful method for the visual confirmation of the morphology and physical state of the surface [44]. SEM has been used, for example, for determining the surface morphology of fungi, and chitin and chitosan in crab shells [340–342], for examining the morphology of deacetylated chitosan powder and chitosan films [140], and for characterizing the surface of chitosan and various types of crosslinked deacetylated chitin before and after metal binding [90]. Nowadays this technique is applied mostly to the determination of new chitin and chitosan derivatives, for example, to study the microstructure of chitosan(chitin)/cellulose biosorbents [49], to characterize the novel nanocomposite scaffold of chitosan and bioactive glass ceramic nanoparticles [343], to characterize the morphology of the surface and the cross-section of chitosan-silica hybrid membranes [344] and to investigate the morphology of vulcanized natural rubber/chitosan blends [91].

Morphological analysis of chitin/chitosan and their derivatives has also been carried out using the SEM-TEM system, for instance, to reveal the structural features of a chitosan/hydroxyapatite nanocomposite [345] or to observe the changes in morphology during the *N-*deacetylation of chitin nanowhiskers to a chitosan nanoscaffold [115].

EDAX is used, among other things, to determine the metal uptake mechanism on chitosan, a complex phenomenon involving nodule formation on the polymer surface, ion adsorption and ion absorption [346]. This technique is also applied to determine the porosity of chitosan beads and membranes, and the diffusion of metal ions through them [44].

Another spectroscopic technique applied to chitin and chitosan investigations is Inductively Coupled Plasma Spectroscopy [ICP] [49]. This has been used to determine the amount of metal ions adsorbed onto chitosan/chitin cellulose biosorbents. The adsorption capacity of these chitosan biosorbents was found to be Cu(II) (0.417 mmol/g) > Zn(II) (0.303 mmol/g) > Cr(VI) (0.251 mmol/g) > Ni(II) (0.225 mmol/g) > Pb(II) (0.127 mmol/g). Inductively-coupled plasma spectrometry was also used for the same reason by Gamage in 2007 [347].

CD spectroscopy measures differences in the absorption of left-handed polarized light *versus* right-handed polarized light that arise due to structural asymmetry [348]. A CD spectrometer records this phenomenon as a function of wavelength. Kittur *et al.* [349] used CD to investigate the degree of *N-*acetylation of low-molecular-weight chitosan (LMWC) prepared from pectolytic hydrolysates of chitosan. Circular dichroism spectra showed a decrease in the segment of *N-*acetylated glucosamine units in LMWC.

In general spectroscopic techniques are very useful and important in the structural analysis of chitin, chitosan and their derivatives, although we need to mention that there are many other non-spectroscopic analytical methods that are routinely used in such studies: viscosity measurements, thermogravimetric analysis (TGA), differential scanning calorimetry (DSC), isothermal titration calorimetry (ITC), cyclic voltamperometry, high-performance liquid chromatography (HPLC) and size exclusion chromatography (SEC).

## 3. Conclusions

Chitin and chitosan are natural aminopolysaccharides with unique structures and interesting properties such as biocompatibility and biodegradability; they are non-toxic, have a wide range of applications, and the raw material sources for their production are unlimited. Chemical modification of these polymers results in improved solubility in water or organic solvents, which enhances their biological activities and favors the continuous development of their applications as new functional biomaterials with excellent potential in various fields. In order to acquire a deeper understanding of the mechanism of these properties, it is necessary for chitin/chitosan and their derivatives to be structurally and physicochemically well characterized. Knowledge of the microstructure of these compounds is essential for an understanding of structure–property–activity relationships. Although spectroscopic methods are of great use to scientists working in the chitin/chitosan field, many researchers lack specialist knowledge of these techniques. This paper focuses on the practical aspects of using the five spectroscopic methods most often applied – X-ray spectroscopy, infrared (IR) and UV-Vis-spectroscopy, mass spectrometry (MS) and nuclear magnetic resonance spectroscopy (NMR-spectroscopy) – in the structural investigation and physicochemical characterization of chitin, chitosan and their derivatives. The application of other spectroscopic methods is also discussed. This paper provides hands-on information about these valuable research tools, emphasizing practical aspects such as sample preparation or typical measurement conditions, their limitations and advantages, and interpretation of results. The usefulness of these methods in establishing and confirming molecular structures, determining physicochemical parameters such as the degree of *N*-acetylation, pattern of *N*-acetylation, degree of polymerization, molecular weight, crystallinity, the sequence or degree of substitution, as well as in monitoring reactions, controlling the purity of these compounds and in characterizing their intra- and intermolecular interactions, is discussed. It should also be mentioned that the structural analysis of chitin/chitosan and their derivatives is based on the application of at least a few different types of spectroscopic methods during the same study. The main reason for this is the possibility of obtaining different but compatible and complete information about the structure and physicochemical properties of these compounds, which is impossible using only one spectroscopic technique. Continuous improvements in sampling techniques, analysis software and instrumentation hardware, mean that spectroscopic methods have revolutionized the chemical and physicochemical characterization of chitin/chitosan samples, and are now routinely used by scientists working in the chitin/chitosan field.

## Figures and Tables

**Table 1:** MS-analyzed chitooligosaccharides obtained after depolymerization using different enzymes.

Enzyme	DP analysis MS method	Chitin/chitosan polymerization degree DP	Chitooligosaccharide applicability	Ref.
Papain	MALDI-TOF	DP 2–DP 8	Antibacterial activity against *Bacillus cereus* and *Escherichia coli*	[222]
Immobilized Papain	MALDI-TOF	DP 3–DP 7	Comparing the depolimerization efficiency between free and immobilized papain	[259]
Pronaze	MALDI-TOF	DP 2–DP 9	Antibacterial activity against *Bacillus cereus* and *Escherichia coli*	[222]
Cellulast (Novozymes)	MALDI-TOF	DP 2–DP 8	Induction of defence response of Oryza sativa L. against Pyricularia grisea (Cooke) Sacc.	[258]
Isozyme of pectinase	MALDI-TOF FAB-MS	DP 2–DP 6	Antimicrobial activity *Bacillus cereus* and *Escherichia coli*	[250]
Chitinase	MALDI-TOF	Q1: DP 3–DP 8 Q2: DP 2–DP 12 Q3: DP 2–DP 10	Affect on fungal (alternaria alternate, *Rhisopus stolnifer, Botrytis cinera, Penicillinum expansum*) growth rate	[262]
Chitosanase	MALDI-TOF	DP 2–DP 8	Investigations of prebiotic effect on the *Bifidobacterium bifidum* and Lactobaccillus sp.	[260]
Chitinase	LC-ESI-MS	DP 2–DP 6	Studies of mechanism of bonding COS to enzyme helpful in the drug-screening program (for drugs in allergic asthma)	[244]

**Table 2:** MS applications in investigations of chitin/chitosan derivatives.

Chitin and chitosan derivative	MS method	Chitooligosaccharide derivative applicability	Ref.
Copper-chitooligosaccharides complexes	ESI-MS, ESI-MS/MS (triple quadrupole, CAD –colision activated dissociation)	Metal-ligand associations studies	[232]
Lipo-chitin oligosaccharides	ESI-MS (quadrupole), FAB-MS, CID-MS/MS (QTof)	Structural studies of lipo-chitin oligosaccharides isolated from bacteria and their role as signal molecules in symbiosis	[247,252]
Products of electrochemical reaction between caffeic acid and glucosamine	ESI-MS	Studies of chitosan–coated electrodes for bimodal sensing	[236]
Methacrylated chitoligosaccharides	MALDI-TOF	Production of biodegradable biopolymers	[225]
Chitosan/tripolyphosphate nanoparticles	ToF-SIMS	Studies of nanoparticles as drug delivery system	[231]
Catechin-modified chitosan	ESI-MS	Creating polymers for technical applications	[235]
Benzenesulfony chitosan, Dinitrobenzenesulfonyl chitosan	MALDI-TOF	Implications for drug detoxification	[265]
Chitosan-g-PEG=X (where X-Man, cholesterol, coumarin, biotin)	MALDI-TOF	Producing copolymers used in active targeting and antiadhesive therapy	[224]
Multilayers consisting of: chitosan, hyaluronan, and poyethyleneimine	ToF-SIMS	Bioactive coating of endovascular stent	[230]
Dodecyl galate (DDG)-chitosan	FAB-MS, ESI-MS	Peroxidaze catalyzed production of biopolymers	[266]
